# MicroRNA-146b promotes adipogenesis by suppressing the SIRT1-FOXO1 cascade

**DOI:** 10.1002/emmm.201302647

**Published:** 2013-09-06

**Authors:** Jiyun Ahn, Hyunjung Lee, Chang Hwa Jung, Tae Il Jeon, Tae Youl Ha

**Affiliations:** 1 Metabolism and Nutrition Research Group, Korea Food Research InstituteSeongnam, Korea; 2 Division of Food Biotechnology, University of Science and TechnologyDaejeon, Korea; 3 Division of Animal Science, University of Chonnam National UniversityGwangju, Korea

**Keywords:** adipogenesis, fat mass, microRNA-146b, obesity, SIRT1

## Abstract

Sirtuin 1 (SIRT1) plays a critical role in the maintenance of metabolic homeostasis and promotes fat mobilization in white adipose tissue. However, regulation of SIRT1 during adipogenesis, particularly through microRNAs, remains unclear. We observed that miR-146b expression was markedly increased during adipogenesis in 3T3-L1 cells. Differentiation of 3T3-L1 was induced by overexpression of miR-146b. Conversely, inhibition of miR-146b decreased adipocyte differentiation. Bioinformatics-based studies suggested that SIRT1 is a target of miR-146b. Further analysis confirmed that SIRT1 was negatively regulated by miR-146b. We also observed that miR-146b bound directly to the 3′-untranslated region of SIRT1 and inhibited adipogenesis through SIRT1 downregulation. The miR-146b/SIRT1 axis mediates adipogenesis through increased acetylation of forkhead box O1 (FOXO1). Expression of miR-146b was increased and SIRT1 mRNA subsequently decreased in the adipose tissues of diet-induced and genetically obese mice. Furthermore, *in vivo* knockdown of miR-146b by a locked nucleic acid miR-146b antagomir significantly reduced body weight and fat volume in accordance with upregulation of SIRT1 and subsequent acetylation of FOXO1. Therefore, the miR-146b/SIRT1 pathway could be a potential target for obesity prevention and treatment.

## INTRODUCTION

Obesity is an energy balance disorder in which nutrient intake chronically exceeds energy expenditure, resulting in the accumulation of white adipose tissue (Hofbauer, 2002). Obesity is frequently associated with insulin resistance and the metabolic syndrome, which is characterized by type 2 diabetes, hypertension, hyperlipidemia and atherosclerosis (Kahn & Flier, 2000). Epidemiological studies suggest that obesity accelerates atherosclerosis (McGill et al, 2002) and is an important predictor of cardiovascular disease (Hubert et al, 1983; Rimm et al, 1995).

Increased adiposity is due to increases in the number and size of adipocytes, which leads to increased body fat and metabolic consequences (Flier, 2004). Adipocytes induce insulin resistance by promoting lipotoxicity and modulating adipokine secretion. Therefore, a thorough understanding of the mechanisms that regulate adipogenesis could have clinical relevance in preventing and treating obesity and the metabolic syndrome.

MicroRNAs (miRNAs) are highly conserved, small non-coding RNAs that regulate gene expression at the post-transcriptional level (Bartel, 2004; Lagos-Quintana et al, 2001; Lee & Ambros, 2001) by binding to complementary sites on target transcripts. Thus, miRNAs are important modulators of developmental and physiological processes, including energy homeostasis, lipid metabolism, pancreatic β-cell development, adipogenesis and weight gain due to a high fat diet (Lin et al, 2009; Takanabe et al, 2008; Wienholds & Plasterk, 2005; Xu et al, 2003). Recently, miRNAs in adipocytes have been shown to alter cell proliferation (the miR-24-1, miR-31 and miR-17-92 cluster) (Sun et al, 2009; Wang et al, 2008), repress Wnt signalling (miR-8) (Kennell et al, 2008), or repress peroxisome proliferator-activated receptor γ (PPARγ; miR-27a, miR-27b and miR-130) (Karbiener et al, 2009; Kim et al, 2010; Lin et al, 2009).

Sirtuin 1 (SIRT1) is most homologous to yeast silent information regulator 2 and primarily localizes to the nucleus. SIRT1 catalyses NAD^+^-dependent protein deacetylation and plays important roles in metabolic homeostasis, including stress resistance, energy metabolism and differentiation (Lagouge et al, 2006). SIRT1 delays adipogenesis in 3T3-L1 cells by inhibiting PPARγ and stimulating lipolysis, which results in reduced fat (Picard et al, 2004). Despite the extensive study of SIRT1 function and its beneficial effects on physiological functions, the mechanisms regulating SIRT1 in adipogenesis remain unclear.

In the present study, we measured changes in miRNA expression during differentiation. We identified miR-146b among the miRNAs whose expression levels changed, because it was robustly upregulated in differentiated cells and played a key role in the adipogenesis of 3T3-L1 cells. Furthermore, we demonstrated that SIRT1 was repressed by miR-146b. We propose a pathway in which miR-146b represses SIRT1-mediated deacetylation of forkhead box O1 (FOXO1) during adipogenesis. We observed a positive correlation between adiposity and expression of miR-146b in obese mice. Furthermore, we demonstrated that systemic knockdown of miR-146b reduced body weight and fat mass. In conclusion, these results suggest that miR-146b is a new SIRT1 inhibitor and potential molecular target for the development of novel therapeutic strategies against obesity.

## RESULTS

### miR-146b promotes adipocyte differentiation

**Figure 1:**
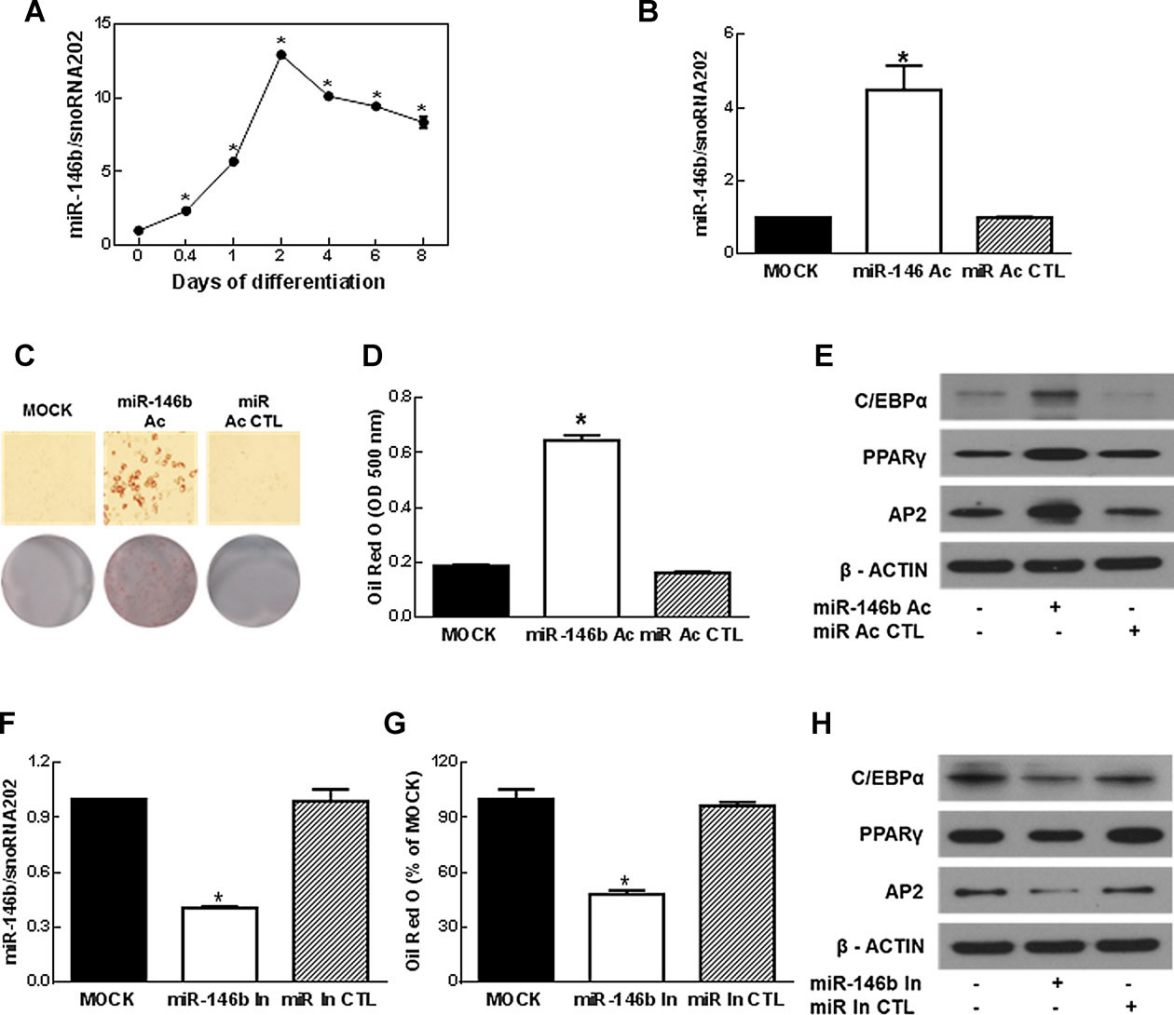
miR-146b induces 3T3-L1 adipocyte differentiation qRT-PCR analysis of miR-146b during 3T3-L1 differentiation (*n* = 4). Values are means ± SD. *p < 0.05 compared with day 0.The effect of miR-146b activator (Ac) on miR-146b expression. Undifferentiated 3T3-L1 cells were transfected with miR-146 Ac or miR Ac control (CTL) and maintained in DMEM supplemented with 10% FBS for 8 d. Expression of miR-146b was calculated relative to MOCK (*n* = 3). Values are means ± SD. *p < 0.05 *versus* miR Ac CTL.The effect of miR-146b Ac on intracellular lipid accumulation in undifferentiated 3T3-L1 cells. Lipid droplets were detected by Oil red O staining.Intracellular lipid accumulation was quantified by measuring optical absorbance at 500 nm (*n* = 3). Values are means ± SD. *p < 0.05 *versus* miR Ac CTL.Western blotting was performed for various adipocyte differentiation markers.The effect of miR-146b inhibitor (In) on miR-146b expression. 3T3-L1 cells transfected with miR-146b or miR In CTL were differentiated according to a standard protocol. Expression of miR-146b was calculated relative to MOCK (*n* = 3). Values are means ± SD. *p < 0.05 *versus* miR In CTL.The effect of miR-146b In on intracellular lipid accumulation in differentiated 3T3-L1 cells. Intracellular lipid accumulation was stained with Oil red O and quantitated by spectrophotometric measurement (*n* = 3). Values are means ± SD. *p < 0.05 *versus* miR In CTL.Western blotting was performed for various adipocyte differentiation markers.

It was previously reported that the miRNA profile changed during adipogenesis (Xie et al, 2009). We measured miRNA expression in preadipocytes and adipocytes by microarray analysis to identify specific miRNAs that modulate adipocyte function (data were now shown). Altered expression levels of miRNAs were confirmed by quantitative real-time polymerase chain reaction (qRT-PCR) and are shown in Supporting Information Table S1. Among the miRNAs whose expression levels changed during differentiation of 3T3-L1 cells, miR-146b was dramatically increased by 9.01-fold. The significant increase in miR-146b expression was maintained during differentiation (Fig 1A). The miR-146b level increased by 12.9-fold within 48 h of differentiation medium exposure and remained high for the remainder of the differentiation time course.

To confirm the role of miR-146b in adipocyte differentiation, we transfected miR-146b activator (Ac) or miR-146b inhibitor (In) and examined differentiation of 3T3-L1 cells. Expression of miR-146b increased when miR-146b Ac was transfected into 3T3-L1 preadipocytes (Fig 1B). Oil red O staining demonstrated that adipogenesis was induced by miR-146b Ac even in the absence of adipogenic stimuli (Fig 1C and D). We measured the protein expression of various adipocyte differentiation markers. Expression levels of two key adipogenic transcription factors, CCAAT/enhancer binding protein alpha (C/EBPα) and PPARγ, and an adipocyte-specific marker, adipocyte fatty acid-binding protein (AP2), were increased after miR-146b Ac transfection (Fig 1E). In contrast, transfection of miR-146b In decreased miR-146b expression and inhibited 3T3-L1 differentiation (Fig 1F and G). We also measured the protein expression of various adipocyte differentiation markers and confirmed decreased expression of adipogenesis markers (Fig 1H).

### miR-146b directly binds and downregulates SIRT1

**Figure 2:**
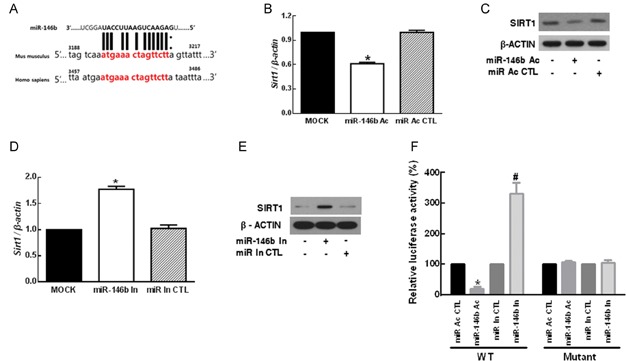
miR-146b negatively modulates SIRT1 The miR-146b sequence and its predicted binding site for the mouse and human SIRT1 mRNA sequences.The effect of miR-146b Ac on the mRNA level of SIRT1 in undifferentiated 3T3-L1 cells. Expression of SIRT1 mRNA was calculated relative to MOCK (*n* = 3). Values are means ± SD. *p < 0.05 *versus* miR Ac CTL.The effect of miR-146b Ac on expression of SIRT1 protein in undifferentiated 3T3-L1 cells.The effect of miR-146b In on SIRT1 mRNA expression in differentiated 3T3-L1 cells. Expression of SIRT1 mRNA was calculated relative to MOCK (*n* = 3). Values are means ± SD. *p < 0.05 *versus* miR In CTL.The effect of miR-146b In on expression of SIRT1 protein in differentiated 3T3-L1 cells.3T3-L1 cells were transfected with wild-type or mutant SIRT1 3′-UTR luciferase constructs and with miR-146b Ac, Ac CTL, miR-146b In or In CTL as indicated (*n* = 5). Values are means ± SD. *p < 0.05 *versus* miR Ac CTL. #*p* < 0.05 *versus* miR In CTL.

Bioinformatics-based prediction of miRNA targets is an alternative approach for identifying the biological functions of individual miRNAs. Gene expression is post-transcriptionally regulated by miRNAs. miRNAs bind to complementary sites on target mRNA sequences and induce cleavage of the transcript or repress translation (Ambros, 2004; Bartel, 2004). We searched for a potential miRNA-146b target gene with miRBase and http://microRNA.org and found a putative miR-146b binding site in the 3′-untranslated region (UTR) of SIRT1 (Fig 2A and Supporting Information Fig S1). Some miRNAs bind to the 5′-UTR of target mRNA sequences (Lytle et al, 2007). We searched for miR-146b binding sites in the 5′-UTR of SIRT1 but did not find any potential sites.

Treatment with miR-146b Ac decreased SIRT1 mRNA and protein expression in comparison with Ac control-treated 3T3-L1 cells (Fig 2B and C). An antisense oligonucleotide against miR-146b induced upregulation of SIRT1 mRNA and protein in differentiated 3T3-L1 cells (Fig 2D and E). To test whether the putative miR-146b binding site in the SIRT1 3′-UTR mediated this repression, we inserted the 3′-UTR transcript or a mutated version of the 3′-UTR into a luciferase expression plasmid and transfected each plasmid into 3T3-L1 cells. In contrast to the reduced luciferase activity observed after miR-146b Ac transfection, mutation of the putative binding site within the SIRT1 3′-UTR abolished the inhibitory effect of miR-146b Ac. The increased luciferase activity caused by miR-146b In was also eliminated in cells that were transfected with the mutant plasmid (Fig 2F).

### miR-146b regulates adipogenesis via modulation of SIRT-1

**Figure 3:**
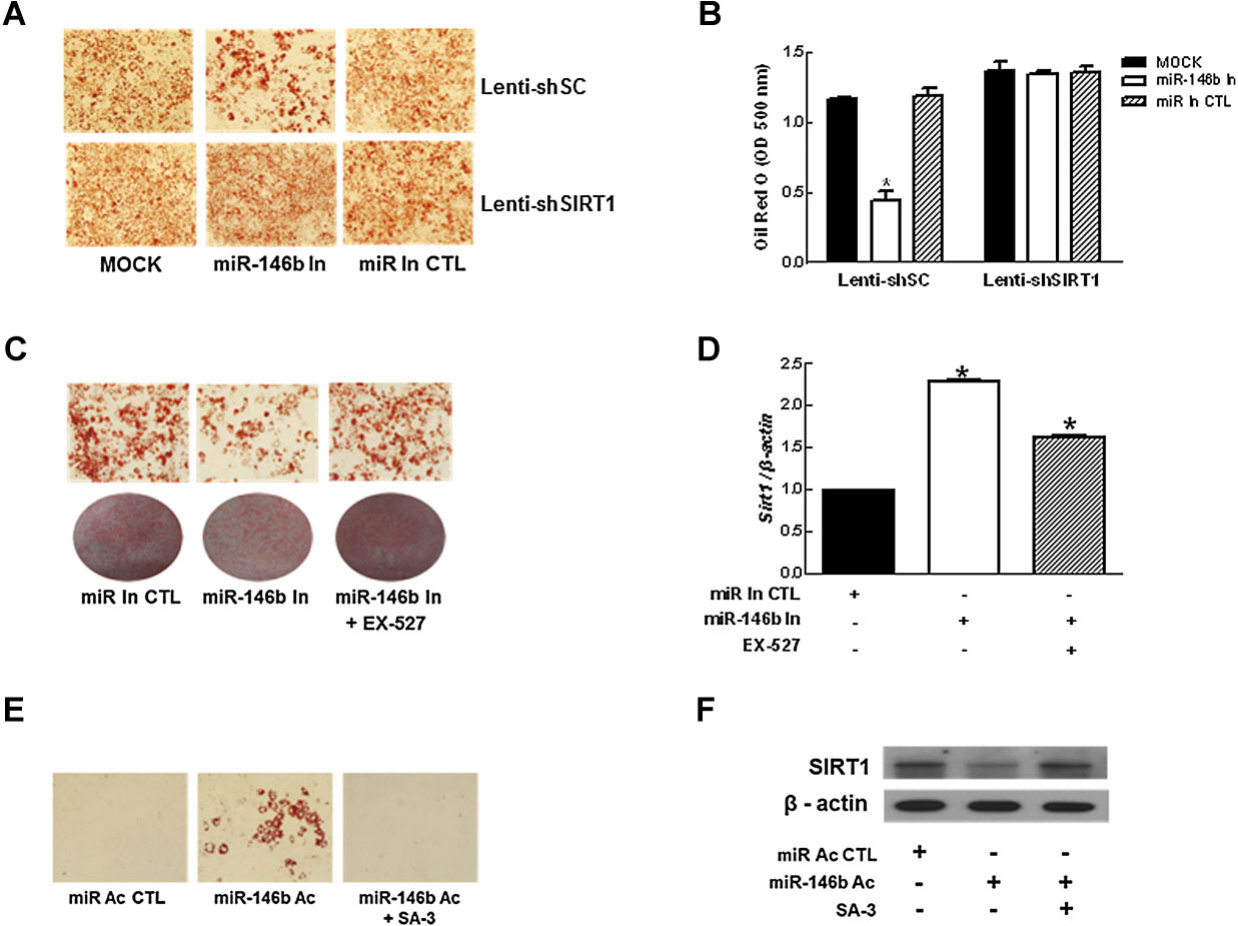
Downregulation of SIRT1 is required for the adipogenic effect of miR-146b Preadipocytes transduced with Lenti-sh NC- or Lenti-sh SIRT1were transfected with miR-146b In or miR In CTL. Cells were stimulated to differentiate after 2 d. Differentiated cells were stained with Oil red O.Intracellular lipid accumulation was stained with Oil red O and quantitated by spectrophotometric measurement (*n* = 3). Values are means ± SD. *p < 0.05 *versus* miR In CTL.The effect of EX-527 on the anti-adipogenic activity of miR-146b In. Cells were pretreated with 10 µM EX-527 for 6 h and then switched to differentiation medium containing 10 µM EX-527. Cells were stained with Oil red O on day 8 of differentiation.The effect of EX-527 on SIRT1 expression in the experiment described in (B). SIRT1 mRNA expression levels were measured by qRT-PCR (*n* = 3). Values are means ± SD. *p < 0.05 *versus* miR In CTL.After 2 d of transfection with miR-146b Ac or miR-Ac CTL, 3T3-L1 cells were maintained in growth medium for 8 d in the presence of 5 µM SA-3. Cells were then stained with Oil red O.The effect of SA-3 on SIRT1 protein expression in the experiment described in (D). Western blot analysis was performed for SIRT1 in total protein extracts.

To investigate the relevance of miR-146b/SIRT1 signalling to adipogenesis, we knocked down SIRT1 expression in 3T3-L1 cells with a lentiviral-based SIRT1 shRNA (Lenti-shSIRT1). Western blotting confirmed reduced SIRT1 expression in cells transduced with Lenti-shSIRT1, whereas no reduction was detected in cells transfected with control shRNA (Lenti-shSC; Supporting Information Fig S2A). As expected, knockdown of SIRT1 increased intracellular fat accumulation (Supporting Information Fig S2B and C). Interestingly, miR-146b In did not inhibit adipogenesis in cells in which SIRT1 had been knocked down, despite marked downregulation of miR-146b (Fig 3A, B and Supporting Information Fig S2D). Similar effects were observed after treating cells with EX-527, a cell-permeable SIRT1 inhibitor (Fig 3C and Supporting Information Fig S3A). Treatment with EX-527 did not affect expression of miR-146b but did reduce SIRT1 mRNA levels (Fig 3D and Supporting Information Fig S3B).

Conversely, treatment with SA-3, an SIRT1 activator, eliminated the pro-adipogenic effect of miR-146b Ac without changing miR-146b expression levels (Fig 3E and Supporting Information Fig S3C). We confirmed that SA-3 recovered SIRT1 at the transcriptional and translational levels (Fig 3F and Supporting Information Fig S3D).

### FOXO1 is a downstream target of the miR-146b/SIRT1 pathway

**Figure 4:**
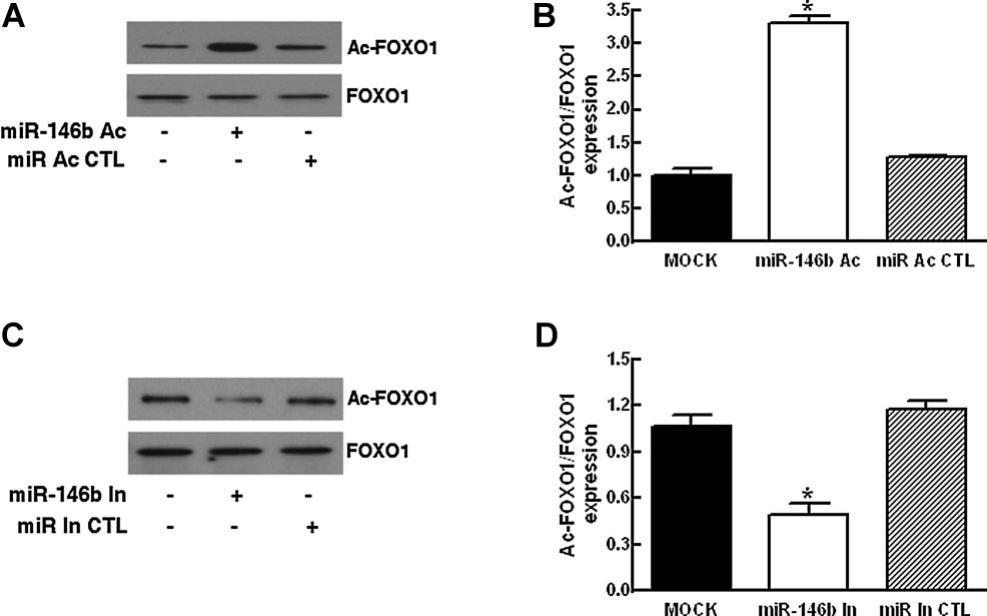
miR-146b/SIRT1 pathway controls the deacetylation of FOXO1 The effect of miR-146b Ac on acetylation of FOXO1 in undifferentiated 3T3-L1 cells. Total and acetylated FOXO1 (Ac-FOXO1) was detected by Western blotting.Ac-FOXO1/FOXO1 ratio was based on densitometric quantifications of Ac-FOXO1 and total FOXO1 levels on Western blots (*n* = 3). *p < 0.05 *versus* miR Ac CTL.The effect of miR-146b In on acetylation of FOXO1 in differentiated 3T3-L1 cells. Total and Ac-FOXO1 was detected by Western blotting.Ac-FOXO1/FOXO1 ratio was based on densitometric quantifications of Ac-FOXO1 and total FOXO1 levels on Western blots (*n* = 3). *p < 0.05 *versus* miR In CTL.

SIRT1 promotes gene transcription by deacetylating specific transcription factors, including FOXO1, a negative regulator of adipogenesis (Armoni et al, 2006). To further investigate the mechanism by which the miR-146b/SIRT1 axis modulates adipogenesis, we analysed the effect of miR-146b on the acetylation of FOXO1. Activation of miR-146b increased the expression of acetylated-FOXO1 (Ac-FOXO1) and induced the rise of Ac-FOXO1/FOXO1 ratio (Fig 4A and B).

Conversely, miR-146b In decreased the expression of Ac-FOXO1 and induced the drop of Ac-FOXO1/FOXO1 ratio (Fig 4C and D). Thus, miR-146b induced adipogenesis by inhibiting SIRT1-mediated deacetylation of FOXO1.

### Silencing of miR-146b ameliorates obesity

To study the role of miR-146b in the development of obesity, we compared miR-146b expression levels in adipose tissues from obese mice and matching control mice. The body weights and fat mass of obese mice were significantly higher than those of control mice (Supporting Information Table S2). As shown in Supporting Information Fig S4A, B and C, we observed a substantial 3.9-, 1.9- and 3.4-fold upregulation of miR-146b in ob/ob, db/db, and diet-induced obese mice, respectively, in comparison with corresponding control mice. Conversely, the expression of SIRT1 was significantly decreased in genetically- and diet-induced obese mice. These data suggested that miR-146b was upregulated with subsequent downregulation of SIRT1 in the white adipose tissue of obese mice.

Next, we investigated the effect of reduced miR-146b expression on body weight and adiposity of fat mice. We knocked down miR-146b expression *in vivo* by injecting a locked nucleic acid (LNA)-miR-146b antagomir (LNA-miR-146b) *versus* LNA-scrambled negative control (LNA-scramble) in obese mice that were fed a high-fat diet. In order to test the inhibitory effects of LNA-miR-146b, we analysed miR-146b expression in various tissues from LNA-miR-146b-injected mice. We found that administration of LNA-miR-146b resulted in effective silencing of miR-146b (Supporting Information Fig S5A). Injection of LNAs did not alter serum levels of AST and ALT (Supporting Information Fig S5B and C).

**Figure 5:**
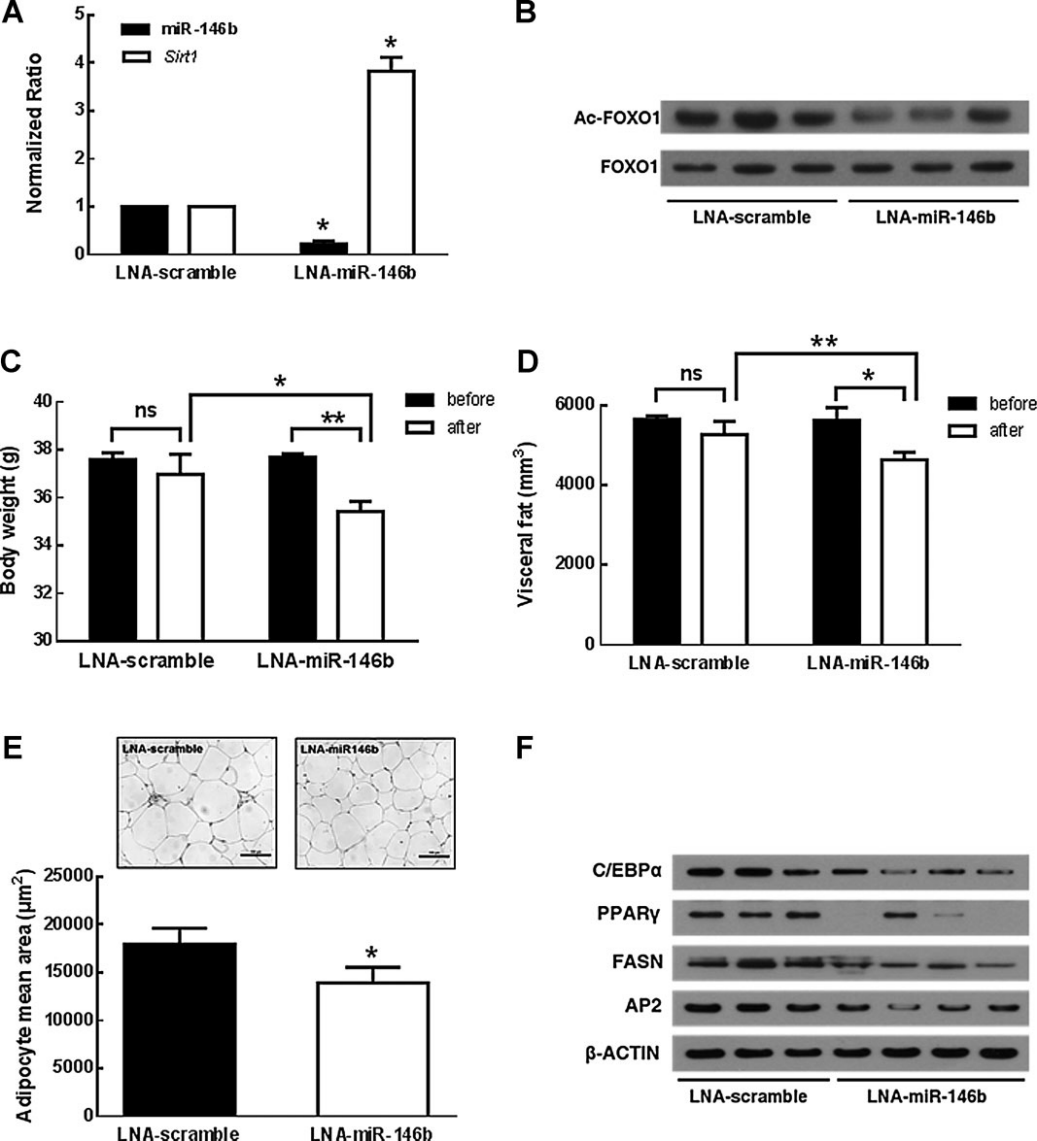
Silencing of miR-146b ameliorates obesity through upregulation of SIRT1 Knockdown of miR-146b by LNA-miR-146b antagomir (LNA-miR-146b) and the subsequent increase in SIRT1 expression were confirmed through qRT-PCR analysis in the epididymal fat tissue of obese C57BL/6J mice. Mice were intraperitoneally injected with 25 mg/kg/d LNA-miR-146b or LNA-scramble for 3 consecutive days and sacrificed 72 h after the last injection (*n* = 5). Values are means ± SEM. *p < 0.05 *versus* LNA-scramble.The effect of miR-146b knockdown by LNA-miR-146b on FOXO1 acetylation. Total and Ac-FOXO1 was detected by Western blotting.Knockdown of miR-146b by LNA-miR-146b induced weight loss in mice. Body weight was measured 72 h after the last injection (*n* = 5). Values are means ± SEM. ns, not significant; *p < 0.05, **p < 0.01.Knockdown of miR-146b by LNA-miR-146b reduced visceral fat volume. CT images were acquired from anaesthetized mice and analysed to calculate visceral fat mass (*n* = 5). Values are means ± SEM. ns, not significant; *p < 0.05, **p < 0.01.Knockdown of miR-146b by LNA-miR-146b induced hypotrophy of white adipose tissues. Perirenal fat fads were stained with H&E (left), and the mean sizes of adipocytes were calculated (right) (*n* = 5). Scale bar = 100 µm. Values are means ± SEM. *p < 0.05 *versus* LNA-scramble.Western blot analysis on adipogenesis-related markers of white adipose tissue from LNA-146b-injected mice. Expressions of C/EBPα, PPARγ, fatty acid synthase and AP2 of perirenal fat tissues were measured. β-actin was used as a loading control.

**Figure 6:**
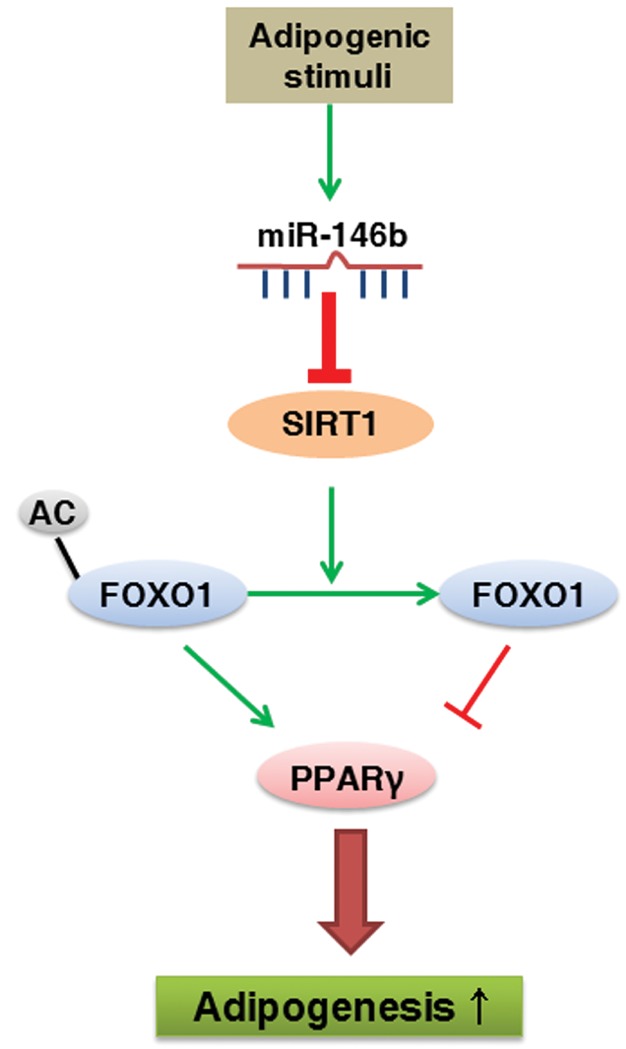
Schematic representation of the pro-adipogenic effect of miR-146b Adipogenic stimuli induce expression of miR-146b. Increased miR-146b binds directly to SIRT1 and downregulates SIRT1 expression. SIRT1 downregulation reduces deacetylation of FOXO1. As a result, FOXO1-mediated inhibition of PPARγ transcription is diminished and adipocyte differentiation is stimulated.

LNA-miR-146b knocked down miR-146b with subsequent upregulation of SIRT1 in perirenal adipose tissues (Fig 5A). LNA-miR-146b treatment also increased deacetylation of FOXO1 (Fig 5B and Supporting Information Fig S6A). We observed that LNA-miR-146b effectively reduced body weight and white adipose tissue weight (Fig 5C and Supporting Information Fig S5B). Computed tomography (CT) analysis demonstrated that the volume of visceral fat was reduced by 17.5% after injection of LNA-miR-146b (Fig 5D). Histological examination clearly showed a decrease in adipocyte cell size after treatment with LNA-miR-146b in comparison to LNA-scramble (Fig 5E). In addition, whole body mass and total body fat were significantly decreased after silencing miR-146b (Supporting Information Fig S6C and D). Western blot analysis showed LNA-miR-146b administration markedly decreased adipogenesis-related transcription factors and adipocyte markers (Fig 5F). Dyslipidemia and hepatic steatosis were effectively improved by LNA-miR-146b injection (Supporting Information Table S3 and Fig S7A-B). We observed the subsequent upregulation of SIRT1 in livers from the LNA-miR-146b injected mice (Supporting Information Fig S7C and D). Moreover, glucose- and insulin tolerance test demonstrated the amelioration of insulin resistance by LNA-miR-146b antagomir (Supporting Information Fig S8A and B). The fasting glucose, insulin and leptin levels were significantly reduced in miR-146b knockdown mice (Supporting Information Table S4). HOMA-IR, an indicator for insulin resistance, was also effectively decreased by LNA-miR-146b administration. Together, these data demonstrated that downregulation of miR-146b ameliorated adiposity and reduced body weight. The proposed mechanism by which miR-146b regulates adipogenesis is summarized in Fig 6.

## DISCUSSION

Obesity is characterized by increased lipid storage in adipocytes and an increased number of adipocytes. Adipocytes are derived from a pool of existing preadipocytes, which are ready to differentiate in response to an appropriate signal. This observation has caused intense research into the mechanisms regulating adipocyte development (Ntambi & Young-Cheul, 2000).

Several miRNAs, including miR-27a, miR-130 and miR-143, were previously shown to regulate adipogenesis. miR-27a and miR-130 suppress adipogenesis by inhibiting PPARγ (Kajimoto et al, 2006), whereas miR-143 induces adipogenesis by downregulating ERK5 (Esau et al, 2004). These results suggest that miRNAs play important roles in regulating adipocyte differentiation.

In the present study, we identified a new pro-adipogenic miRNA, miR-146b. Gain- or loss-of-function studies showed that miR-146b promoted adipocyte differentiation. A targeting study demonstrated that miR-146b directly bound to the 3′-UTR of SIRT1 and downregulated SIRT1 at the transcriptional and translational levels. Experiments in which SIRT1 was silenced, inhibited by EX-527, or activated by SA-3 demonstrated that upregulation of SIRT1 abolished miR-146b-induced adipogenesis.

The role of miR-146b has been studied in various cancer cell lines. Overexpression of miR-146b impedes invasion and migration of U373 (Xia et al, 2009) and U87-MG (Katakowski et al, 2010) glioblastoma cells and MDA-MB-231 breast cancer cells (Hurst et al, 2009). Conversely, an oncogenic role for miR-146b in thyroid cancer (Geraldo et al, 2012) and lung cancers was reported (Patnaik et al, 2011). Furthermore, miR-146b negatively regulates the nuclear factor-kB pathway (Bhaumik et al, 2008) in breast cancer cells. Similarly, decreased expression of miR-146b in the monocytes of obese subjects is associated with inflammation (Hulsmans et al, 2012). Although miR-146b is regarded as a relevant diagnostic marker for certain types of cancer, potential roles in adipogenesis and hypertrophy of adipose tissue have not yet been demonstrated.

SIRT1 promotes gene transcription by deacetylating specific transcription factors, including FOXO1 (Daitoku et al, 2004) and PGC1α (Nemoto et al, 2005). The role of SIRT1 as a regulator of metabolic homeostasis has been investigated and reviewed extensively (Silva & Wahlestedt, 2010). SIRT1 is downregulated in adipocytes during adipogenesis (Jing et al, 2007). SIRT1 inhibits adipogenesis by interacting with the PPARγ co-repressors, nuclear receptor co-repressor (N-CoR) and silencing mediator of retinoid and thyroid hormone receptors (SMRT), thereby inhibiting PPARγ (Picard et al, 2004). However, the mechanisms regulating SIRT1 during adipogenesis have not been determined. In this study, we demonstrate that miR-146b is an upstream negative regulator of SIRT1 during adipocyte differentiation.

There are several reports concerning the regulation of SIRT1 by miRNAs. miR-34a was first identified as a post-transcriptional regulator of SIRT1 during the regulation of apoptosis and fatty liver disease (Lee et al, 2010; Yamakuchi et al, 2008). miR-132 and miR-199a were shown to downregulate SIRT1 in response to nutritional availability and hypoxia, respectively (Rane et al, 2009; Strum et al, 2009). These observations suggest that SIRT1 regulation is precisely controlled by miRNAs during diverse biological processes.

The FOXO1 transcription factor has an important role in adipogenesis, because it inhibits adipocyte differentiation at an early phase (Dowell et al, 2003; Nakae et al, 2003). FOXO1 interacts with PPARγ and negatively regulates its transcriptional activity in adipose tissue. FOXO1 binds to the PPARγ promoter region and suppresses PPARγ expression (Armoni et al, 2006). In addition, FOXO1 can upregulate p21 expression and inhibit clonal expansion (Nakae et al, 2003). Acetylation of FOXO1 attenuates DNA-binding and represses its transcriptional activity (Matsuzaki et al, 2005). SIRT1 regulates FOXO1 transactivation activity by deacetylating three lysine residues within the forkhead DNA binding domain (van der Heide & Smidt, 2005). Similarly, SIRT2 regulates 3T3-L differentiation by regulating FOXO1 acetylation/deacetylation. A miR-146b activator induced acetylation of FOXO1 as a result of SIRT1 downregulation. Conversely, mir-146b inhibition evoked deacetylation of FOXO1 in differentiated adipocytes. We confirmed that PPARγ expression was inversely related to deacetylation of FOXO1. Therefore, these observations led us to conclude miR-146b/SIRT1 pathway regulates adipogenesis via acetylation of FOXO1.

Increased miR-146b levels were observed in the white adipose tissue of obese mice, such as ob/ob, db/db and diet-induced obesity (DIO) mice. This observation is consistent with previous reports (Xie et al, 2009). Upregulation of miR-146b was accompanied by a reduction in SIRT1 levels. Moreover, we observed an *in vivo* knockdown of miR-146b by LNA-miR-146b reduced body weight and adiposity in obese mice along with upregulation of SIRT1. The expressions of adipogenesis related markers were markedly decreased in adipose tissues by knockdown of miR-146b.

Development of oligonucleotides that target disease-associated miRNAs is a promising therapeutic strategy. LNA is a class of bicyclic conformational analogues of RNA that exhibits a high affinity for complementary target RNA sequences, leading to selective inhibition of gene expression (Fluiter et al, 2003). Here, we showed that systemic administration of a 16-nucleotide LNA inhibitor complementary to the 5′-end of miR-146b specifically silenced miR-146b expression *in vivo* without hepatotoxicity. Metabolic disorders including dyslipidemia, hepatic steatosis, and insulin resistance were improved in LNA-146b injected obese mice. Our result is in line with previous study (Li et al, 2011) which reported overexpression of SIRT1 protected insulin resistance and improved metabolic parameters. Overexpression of SIRT1 reduced body weight gain and prevented hepatic steatosis associated with a high-fat diet. These effects are due to the antioxidant and anti-inflammatory activities of SIRT1 (Bordone et al, 2007; Pfluger et al, 2008).

On the contrary, hepatic-specific deletion of SIRT1 shows greater lipid accumulation in the liver as a result of reduced fatty acid oxidation. However, this does not alter serum triglyceride and cholesterol levels (Purushotham et al, 2009). Also, hepatic knockout of SIRT1 fails to alter serum insulin and leptin levels and shows normal insulin sensitivity and fuel metabolism in white adipose tissue and muscle and thus does not cause systemic glucose intolerance. These observations suggest that upregulation of hepatic SIRT1 alone is not sufficient to improve systemic insulin resistance, dyslipidemia and hyperinsulinemia. Instead, adipose tissue is important in controlling whole body metabolism by releasing adipokines and storing free fatty acids. Consistently, decrease of SIRT1 in liver and adipocyte effectively reduces white adipose tissue mass and improves glucose tolerance (Erion et al, 2009). Thus, increased adipose mass via hyperplasia and hypertrophy results in the insulin resistance and adipocyte dysfunction (Roberts et al, 2009). Therefore, reduced adiposity through downregulated miR-146b plays a crucial role in attenuating metabolic disorders including insulin resistance and dyslipidemia. Although we found that systemic knockdown of miR-146b effectively reduced body weight and adiposity, further studies are needed to confirm the direct role of miR-146b using adipose tissue-specific knockout of miR-146b.

In summary, our study provides direct evidence that miR-146b induces post-transcriptional SIRT1 silencing, which contributes to adipogenesis. We identified miR-146b as a new regulator of adipogenesis and characterized the miR-146b/SIRT1 pathway as a potential target for preventing and treating obesity. Therefore, miR-146b is an attractive target for new therapies aimed at reducing excess fat.

## MATERIALS AND METHODS

### Cell culture and differentiation

3T3-L1 fibroblasts (ATCC, Manassas, VA) or transduced cells were maintained and differentiated as described previously (Ahn et al, 2010). Briefly, 3T3-L1 cells were maintained in Dulbecco's modified Eagle medium (DMEM) containing 25 mM glucose, 10% calf serum, 100 U/ml penicillin, 100 µg/ml streptomycin and 2 mM l-glutamine. For differentiation, cells were plated at a density that allowed them to reach confluence in 3 days (d). At this point (day 0), cells were switched to DMEM supplemented with 10% fetal bovine serum (FBS) with MDI (0.25 µM dexamethasone, 0.25 mM 1-methyl-3-(2-methylpropyl)-7H-purine-2,6-dione [IBMX] and 1 µg/ml insulin). Dexamethasone and IBMX were removed on day 3, and cells were cultured in insulin-containing media for two additional days. Cells were incubated in 10% FBS/DMEM for 4 additional days, at which time (day 8) 90% of the control cells were mature adipocytes that had accumulated fat droplets. After differentiation, cells were stained with Oil red O (Sigma–Aldrich, St. Louis, MO, USA). Images were collected with an Olympus microscope (Tokyo, Japan). Stained oil droplets were dissolved in isopropanol and quantified by measuring optical absorbance at 500 nm.

**The paper explained**

PROBLEM:

Obesity is rapidly becoming a worldwide epidemic and is associated with increased risks of developing cardiovascular disease and types 2 diabetes. An increase in adipose tissue mass occurs due to increased cell size (hypertrophic obesity) and cell number (hyperplastic obesity). The main function of adipocytes is to store triglycerides during periods of energy excess and to mobilize energy during deprivation. Thus, understanding the mechanisms that regulate fat cell development is of great clinical importance, as this will allow new prevention and treatment strategies to be designed for metabolic diseases. Recently, microRNAs (miRNAs) have been shown to play roles in metabolic disease development. Dysregulation of miRNAs contributes to the pathogenesis of obesity-related complications. However, functional studies that identify direct molecular targets and reveal signalling pathways that regulate adipogenesis remain to be performed. Several lines of evidence have shown that SIRT1 protects against metabolic damage. Extensive studies of SIRT1 function and its beneficial effects have been reported. However, the mechanisms by which SIRT1 expression is regulated in normal conditions and metabolic disease states remain unclear.

RESULTS:

Expression of miRNA-146b, a well-known inhibitor of glioma cell migration, was increased during adipogenesis. Suppression of miR-146b inhibited adipogenesis, whereas miR-146b overexpression induced differentiation even in the absence of adipogenic stimuli. We identified SIRT1 as a direct functional target of microRNA-146b and confirmed signalling through the miR-146b/SIRT1/FOXO1 pathway. Upregulation of miR-146b with subsequent downregulation of SIRT1 was observed in the adipose tissue of obese mice, such as DIO, ob/ob and db/db. In addition, systemic silencing of miR-146b reduced weight gain and fat mass and improved hepatic steatosis.

IMPACT:

We show that miR-146b plays an important role in adipogenesis by downregulating SIRT1 and subsequently increasing acetylated FOXO1. Inhibition of miR-146b reduced body weight and fat mass in obese mice. Considering the rapidly increasing prevalence of obesity and the metabolic syndrome, miR-146b is a potential new target for controlling adipose tissue mass and function.

### Quantitative real-time polymerase chain reaction

Total RNA was isolated with a NucleoSpin® RNA II kit (Macherey-Nagel, Duren, Germany). Total RNA was reverse transcribed with the TaqMan™ MicroRNA reverse transcription kit and analysed by real-time PCR with the TaqMan™ MicroRNA assay kit (Applied Biosystems, Foster City, CA, USA) to quantify mature miRNA. Expression of miRNA was normalized to endogenous snoRNA202. For the mRNA expression assay, cDNA was prepared as previously described (Chomczynski & Sacchi, 1987). qRT-PCR was performed with SYBR® Green PCR Master Mix in a StepOnePlus Real-Time PCR system (Applied Biosystems). The level of each mRNA was normalized to β-actin. The PCR primer sequences (5′ → 3′) were SIRT1, forward: AGA ACC ACC AAA GCG GAA A, reverse: TCC CAC AGG AGA CAG AAA CC; FOXO1, forward: CCC AGG CCG GAG TTT AAC C, reverse: GTT GCT CAT AAA GTC GGT GCT; β-actin, forward: AAT ACC CCA GCC ATG TGT GT, reverse: ATG GGC ACT GTG TGT GAC C.

### Functional study of miRNA

We examined the effect of gain- or loss-of-function of miR-146b on the differentiation of 3T3-L1 cells. 3T3-L1 cells were transfected with a mirVana™ miR-146b activator (Ac) or an Ac control (CTL; Life Technologies, Grand Island, NY, USA) and maintained in DMEM supplemented with 10% FBS for 8 d. Alternatively, 3T3-L1 cells were transfected with a mirVana™ miR-146b inhibitor (In) or an In CTL (Life Technologies) 2 d before differentiation according to a standard protocol. Oligonucleotides (10 nM) were transfected into cells with Lipofectamine™ RNAiMAX (Invitrogen, Carlsbad, CA, USA). Overexpression or inhibition of miR-146b expression was verified by qRT-PCR as described above. The negative Ac and In CTLs have unique sequences designed that they do not target any human, mouse, or rat genes and are validated not to produce identifiable effects on known miRNAs functions.

### Western blot

Cells and adipose tissues were lysed with RIPA buffer. Western blot analysis was performed as previously described (Ahn et al, 2010). Blots were probed with primary antibodies against C/EBPα, PPARγ, β-ACTIN (from Cell Signalling, Danvers, MA, USA), AP2, SIRT1, FOXO1 and acetyl-FOXO1 (from Santa Cruz Biotechnology Inc., Santa Cruz, CA, USA).

### 3′-UTR luciferase reporter assays

The SIRT1 3′-UTR was confirmed to act as a binding site for miR-146b with the pMIR-REPORT™ System (Ambion, Austin, TX, USA). The cDNA fragment corresponding to the 3′-UTR of SIRT1 was inserted at the designated multiple cloning site downstream of the firefly luciferase reporter gene, which was controlled by the CMV promoter of the pMIR-REPORT™ miRNA expression reporter vector. Transfection efficiency was normalized with the pMIR-REPORT™ β-galactosidase reporter control vector. 3T3-L1 cells were co-transfected with Fugene 6 (Roche Applied Science) and 150 ng of the 3′-UTR luciferase reporter or control vector and 150 ng of miR-146b Ac, Ac CTL, miR-146b In or In CTL vector. Luciferase activity was measured using the Dual-Light® System (Applied Biosystems). Luciferase activity was normalized to the corresponding β-galactosidase activity 48 h after transfection and was plotted as a percentage of the mock transfection (MOCK).

### Lentiviral transduction of cells

3T3-L1 cells were transduced with a lentiviral-based SIRT1 short hairpin RNA (shRNA; Genepharma, Shanghai, China) according to the manufacturer's protocol for LentiSuite™ (System Bioscience, Mountain View, CA, USA). SIRT1 knockdown was confirmed by immunoblotting with an anti-SIRT1 antibody.

### Modulation of SIRT1 activity

SIRT1 was inhibited by treating with 10 µM 6-chloro-2,3,4,9-tetrahydro-1H-carbazole-1-carboxamide [EX-527] (Enzo Life Sciences, Framingdale, MI, USA) in 3T3-L1 cells that had been transfected with a miR-146b inhibitor. After 6 h of treatment with EX-527, cells were switched to differentiation media, which consisted of DMEM supplemented with 10% FBS and MDI in the presence of 10 µM EX-527. Cells continued to differentiate on day 3 as described above. Differentiated cells were harvested for analysis or stained with Oil red O.

SIRT1 was activated by treating cells with 5 µM SIRT1 Activator 3 (SA-3) (Santa Cruz Biotechnology) after 48 h of transfection with miR-146b Ac. The medium was changed every 2 d. Fresh EX-527 was added with each medium change. Oil red O staining was performed on day 8 to detect intracellular lipid deposits.

### Mouse studies

Studies were performed with 6-week old male C57BL/6J (Orient Bio Inc. Seongnam-si, Korea), ob/ob, and db/db mice (The Jackson Laboratory, Bar Harbor, ME, USA). All animal studies were conducted in accordance with a protocol approved by the Korea Food Research Institute's Institutional Animal Care and Use Committee. The ob/ob and db/db mice were fed a normal diet (LabDiet, Purina, St. Louis, MO, USA) for 6 weeks. Mice were fed either a normal or high-fat diet, in which 45% of total calories were derived from fat, for 8 weeks to establish a DIO model. Mice were sacrificed after a 12-h fast, and epididymal fat pads were collected.

Knockdown of miR-146b was achieved *in vivo* by administering a LNA-miR-146b antagomir or LNA-scrambled control to obese mice that were fed a high-fat diet for 8 weeks. Mice received daily intraperitoneal injections of 25 mg/kg LNA-miR-146b or LNA-scramble dissolved in saline for 3 consecutive days (a total volume of 100 µl). Mice were sacrificed 72 h after the last injection.

### Computed tomography

CT was performed to measure whole body mass, total body fat, and visceral fat volume in live mice. Mice were anaesthetized with Zoletil 50 (Virbac, Carros cedex, France). CT scans were run with eXplore CT120 (GE Healthcare, Buckinghamshire, UK) in fat scan mode (36 mGy, 70 kV and 32 mA). Each scan took 87 s including calibration, and the resolution was 100 µm. Images were analysed with Microview software (GE Healthcare). The Hounsefield units of −400 to 4000 and −400 to −10 were defined as whole body mass and fat mass, respectively, in the histograms of the CT images. Visceral fat distribution was assessed from the diaphragm to the pelvic floor.

### Statistical analysis

Results are expressed as the means ± standard deviation or standard errors of the means. Statistical analyses were performed with GraphPad Prism 6 software (San Diego, CA, USA). Quantitative data among groups was compared by one-way analysis of variance. The Bonferroni post-hoc test was used if analysis of variance indicated significance (*p* < 0.05).

## Supplementary material

As a service to our authors and readers, this journal provides supporting information supplied by the authors. Such materials are peer reviewed and may be re-organized for online delivery, but are not copy-edited or typeset. Technical support issues arising from supporting information (other than missing files) should be addressed to the authors.

**Figure S1.** Target analysis predicts that SIRT1 is a potential target of miR-146b.

MicroRNA target prediction databases, such as http://microRNA.org, showed alignment between miR-146b and SIRT1.

**Figure S2.** Knockdown of SIRT1 increased lipid accumulation in differentiated 3T3-L1 cells.

A. SIRT1 was knocked down by lentiviral shRNA-SIRT1 (Lenti-sh SIRT1) and confirmed by Western blot analysis. Lenti-sh NC, lentiviral shRNA negative control.

B. Preadipocytes transduced with Lenti-sh NC- or Lenti-sh were stimulated to differentiation. At day 8, differentiated cells were stained with Oil red O.

C. Intracellular lipid accumulation was quantified by measuring optical absorbance at 500 nm (*n* = 3). ***p* < 0.01. Values are means ± SD.

D. Preadipocytes that were transduced with Lenti-sh NC or Lenti-sh SIRT1 were transfected with miR-146b In or miR In CTL and stimulated to differentiate. Expression levels of miR-146b and SIRT1 were quantified by qRT-PCR on day 8 of differentiation (*n* = 3). Values are means ± SD. **p* < 0.05 *versus* miR In CTL.

**Figure S3.** SIRT1 mediates the adipogenic effect of miR-146b in 3T3-L1 cells.

A. The effect of EX-527 on the anti-adipogenic activity of miR-146b In. Cells were pretreated with 10 μM EX-527 for 6 h. Media were then replaced with differentiation media containing 10 μM EX-527. Cells were stained with Oil red O on day 8 of differentiation, and lipid droplets were quantified (*n* = 3). Values are means ± SD. **p* < 0.05 *versus* miR In CTL.

B. The effect of EX-527 on miR-146b expression in the experiment described in (A). Expression of miR-146b was measured by qRT-PCR (*n* = 3). Values are means ± SD. **p* < 0.05 *versus* miR In CTL.

C. The effect of SA-3 on miR-146b expression. 3T3-L1 cells were transfected with miR-146b Ac or miR-Ac CTL. After 2 days, cells were maintained in growth media containing 5 μM SA-3 for 8 days. Expression levels of miR-146b were measured by qRT-PCR (*n* = 3). Values are means ± SD. **p* < 0.05 *versus* miR Ac CTL.

D. The effect of SA-3 on SIRT1 mRNA expression in the experiment described in (C). Expression levels of SIRT1 mRNA were measured by qRT-PCR (*n* = 3). Values are means ± SD. **p* < 0.05 *versus* miR Ac CTL.

**Figure S4.** Expression of miR-146b and its direct target, SIRT1 in WAT from various obese mice.

A–C. The increase in miR-146b and the resulting decrease in SIRT1 are correlated with hypertrophy of adipose tissue from obese mice, such as ob/ob (A), db/db (B) and diet-induced obese mice (C). qRT-PCR analysis was performed to measure miR-146b and SIRT1 mRNA expression levels in epididymal adipose tissue (*n* = 5). Values are means ± SEM. **p* < 0.05 *versus* corresponding control mice.

**Figure S5.** Efficacy and toxicity of LNA-miR-146b antagomir.

A. Expression levels of miR-146b were measured by qRT-PCR in various tissues to test the efficacy of LNAmiR-146b antagomir in mice. Expression of miR-146b was compared between mice treated with LNA-miR-146b and those treated with LNA-scrambled negative control (*n* = 5). Values are means ± SEM. EF, epididymal fat pad; PF, perirenal fat; BF, brown fat; SF, subcutaneous fat; SI, small intestine; LI, large intestine.

B, C. Effect of LNA-miR146b injection on AST and ALT, which are biomarkers of liver injury (*n* = 5). Values are means ± SEM.

**Figure S6.** Knockdown of miR-146b reduced adiposity and whole body mass in high fat-fed obese mice.

A. Knockdown of miR-146b by LNA-miR-146b significantly decreased the acetylated FOXO1(Ac-FOXO1)/total FOXO1 ratio of white adipose tissue. Ac-FOXO1/FOXO1 ratio was based on densitometric quantifications of Ac-FOXO1 and total FOXO1 levels on Western blots (*n* = 5). Values are means ± SEM. **p* < 0.05 *versus* LNA-scramble.

B. Knockdown of miR-146b by LNA-miR-146b reduced white adipose fat mass. The weight of perirenal white adipose tissue was measured 72 h after the last injection (*n* = 5). Values are means ± SEM. **p* < 0.05 *versus* LNA-scramble.

C. Administration of LNA-miR-146b inhibitor (LNA-miR-146b) significantly decreased whole body mass (*n* = 5).

Values are means ± SEM. ns, not significant; **p* < 0.05.

D. LNA-miR-146b significantly reduced total body fat mass compared to LNA-scramble (*n* = 5). Values are means ±SEM. ns, not significant; **p* < 0.05.

**Figure S7.** Knockdown of miR-146b improved hepatic steatosis via upregulation of SIRT1 in high fat-fed obese mice.

A. Administration of LNA-miR-146b reduced hepatic hypertrophy (*n* = 5). Values are means ± SEM. **p* < 0.05 *versus* LNA-scramble.

B. Injection of LNA-miR-146b decreased intracellular lipid accumulation in hepatocytes. Hepatic tissues were stained with H&E for histological examination. Scale bar = 100 μm.

C. Immunoblot showing SIRT1 and β-actin expressions.

D. Densitometric analyses for (C). Values are means ± SD. ***p* < 0.01.

**Figure S8.** Knockdown of miR-146b ameliorated insulin resistance in high fat-fed obese mice.

A. Glucose tolerance test in LNA-scramble and LNA-miR146b injected mice (*n* = 6). Values are means ± SEM. **p* < 0.05 *versus* LNA-scramble.

B. Insulin tolerance test in LNA-scramble and LNA-miR146b injected mice (*n* = 6). Values are means ± SEM. **p* < 0.05 *versus* LNA-scramble.

**Table S1.** Changes in the expression levels of various miRNAs after adipogenesis in 3T3-L1.

**Table S2.** Measurement of body weight and white adipose tissue (WAT) weight.

**Table S3.** Serum lipids profile in LNA-injected mice.

**Table S4.** Blood glucose and hormones levels in LNA-injected mice.
