# Supplementary material for: MicroRNA-146b promotes adipogenesis by suppressing the SIRT1-FOXO1 cascade
Source: EMBO Mol Med. 2013 Sep 6;5(10):1602–12. doi: 10.1002/emmm.201302647 (PMC3799582; doi:10.1002/emmm.201302647)
Supplement: Supplementary file 1 [file emmm0005-1602-SD1.pdf]

MicroRNA-146b promotes adipogenesis by suppressing the SIRT1-FOXO1 cascade

Jiyun Ahn, Hyunjung Lee, Chang Hwa Jung, Tae Il Jeon and Tae Youl Ha

Corresponding author: Tae Youl Ha, Korea Food Research Institute

Review timeline:

Submission date:	18 February 2013
Editorial Decision:	20 March 2013
Revision received:	23 June 2013
Editorial Decision:	11 July 2013
Revision received:	31 July 2013
Accepted:	02 August 2013

Transaction Report:

(Note: With the exception of the correction of typographical or spelling errors that could be a source of ambiguity, letters and reports are not edited. The original formatting of letters and referee reports may not be reflected in this compilation.)

Editor: Céline Carret

1st Editorial Decision 20 March 2013

Thank you for the submission of your manuscript to EMBO Molecular Medicine. We have now heard back from the three referees whom we asked to evaluate your manuscript. Although the referees find the study to be of potential interest, they also raise a number of significant concerns that need to be addressed in a major revision of the manuscript.

As you will see from the enclosed reports, all three referees find the topic interesting and the molecular axis novel and relevant to molecular medicine. However, all three reviewers have one important concern: the in vivo analysis of the model is at best superficial and should be greatly improved (at the molecular and physiological levels as suggested) to demonstrate that the pathway effectively regulates adipogenesis in vivo.

In addition, referees 2 and 3 are concerned about the transfection experiment and referee 2 suggests using siRNA or shRNA instead, which we would recommend to do as to rule out any unspecific effect on Sirt1 levels.

Referee 3 would also like to see better quantitation and statistical analysis. Referee 2 is concerned about the western blot in figure 3A. To address this point, I would like to suggest to provide the uncropped and unprocessed scan that we would publish online with the article as supplementary "Source Data" file. We do encourage the publication of source data, particularly for electrophoretic gels and blots, with the aim of making primary data more accessible and transparent to the reader. The PDF files should be labeled with the appropriate figure/panel number, and should have molecular weight markers; further annotation may be useful but is not essential.

In conclusion, I would like to give you the opportunity to revise your manuscript, with the understanding that the referee concerns must be fully addressed and that acceptance of the manuscript would entail a second round of review.

Please note that it is EMBO Molecular Medicine policy to allow a single round of revision in order to avoid the delayed publication of research findings. Consequently, acceptance or rejection of the manuscript will depend on the completeness of your responses included in the next version of the manuscript.

EMBO Molecular Medicine has a "scooping protection" policy, whereby similar findings that are published by others during review or revision are not a criterion for rejection. Should you decide to submit a revised version, I do ask that you get in touch after three months if you have not completed it, to update us on the status.

Please also contact us as soon as possible if similar work is published elsewhere. If other work is published we may not be able to extend the revision period beyond three months.

I look forward to seeing a revised form of your manuscript as soon as possible.

***** Reviewer's comments *****

Referee #1 (Comments on Novelty/Model System):

This ms finds a role for increasing miRNA146b with adipose differentiation (increase miRNA146b, decrease SIRT1, decreased FOXO1 deacetylation). This is interesting and novel. A major gap in the paper is the lack of understanding the effect of miRNA146b knockdown on glucose homeostasis. I would require this before accepting the paper; it will entail a major amount of new experimental work to comply.

Referee #1 (Remarks):

This ms finds that miRNA146b increases with and promotes adipose development, while inhibiting it prevents adipose development. The miRNA binds to the 3'UTR of SIRT1 and manipulating miR146b affects SIRT1 RNA and protein (inc miR will dec SIRT1); miR146b acts via SIRT1 to affect adipose differentiation and FOXO1 is downstream. miR146b is increased in mouse obesity models and reduction of miR146b by LNA reduced adiposity in mice.

The observations are novel and interesting.

1. A major omission in the paper is examination of the effect that the LNA/reduced adiposity has on the physiology of the mice. Are these lean, healthy insulin-sensitive mice? Or are they lipodystrophic, insulin resistant? There is a lot of physiology that could/should be done to address this point. The paper would be hugely improved by inclusion of such data (eg clamp, lipid levels, etc).

Referee #2 (Comments on Novelty/Model System):

The novelty of this work is significantly dampened by previous report that SIRT1 regulates adipogenesis and Sirt1+/- mice have reduction in fat. Identifying SIRT1 as a target of miR-146b is indeed a novel finding but authors did not produce convincing data to support a miR-146b/SIRT1 pathway regulates adipogenesis. This is partly due to the fact that their transfection system significantly affects the expression levels of SIRT1, one of their major targets. In addition, their characterization of miR-146b knockdown mice is quite superficial making it difficult to conclude that 146b regulates adipogenesis in vivo.

Referee #2 (Remarks):

The manuscript by Ahn et al reports that miR-146b level increased during 3T3-L1 differentiation, and inhibition or activation of miR-146b decreased or increased adipogenesis respectively. The authors further identified that miR-146b targets SIRT1 to increase FOXO1 acetylation, which mediates its pre-adipogenic effect. They then went on to suppress miR-146b in mice and demonstrated reduced adiposity and hepatosteatosis. In general, this article addresses a very interesting subject in metabolic regulation but the approach is very limited and the mechanism proposed is only partially supported by the authors' data. Key information is missing for several experiments. Therefore, a significant number of additional experiments and clarifications are necessary for the manuscript to be convincing.

Major issues
1. In Fig. 2C, Sirt1 levels transfected with miR-146b Ac or Ac CTL were both significantly lower than that in mock transfection yet only miR-146b Ac induced adipogenesis as shown in Figure 1C. The authors should test different doses of Ac and CTL to make sure that altered SIRT1 expression and adipogenesis were not caused by the transfection per se. The same problem exists in Figure 2E but it is even more troubling since both transfections here increased SIRT1 expression as compared to MOCK. Therefore it appears that the current experimental system is not suitable for studying the role of SIRT1 in adipogenesis. In stead, a lentivirus or retrovirus based system should be used.

2. Again in Figure 2C, SIRT1 protein level in miR Ac CTL transfection is clearly lower than that in MOCK but their mRNA levels are almost the same as shown in Figure 2B. Is there any post-transcription regulation here?

3. Fig. 3A was clearly generated from two blots based on the misalignment of actin bands and different background so they cannot be compared.

4. Foxo1 protein levels in cell lysate need to be shown before the IP data in Figure 4 can be appropriately interpreted. And this applies to Figure 5 as well.

5. The authors interpret that the reduced adiposity in miR-146b knockdown mice was caused by decreased adipocyte differentiation yet did not show a single adipogenic markers such as PPARgamma or aP2. In fact, SIRT1 has been showed to be able to regulate fatty acid oxidation in the liver. All physiological changes observed in miR-146b knockdown mice can be explained if reduced miR-146b in the liver increases SIRT1 level. All these possibilities have to be addressed before the authors can conclude that miR-146b regulates adipogenesis in vivo.

Minor issues
1. Critical physiological parameters such as the levels of serum lipids, glucose and key metabolic hormones in control and miR-146b knockdown mice need to be provided to establish a clear picture on the physiological changes in these mice.

Referee #3 (Comments on Novelty/Model System):

The authors provided evidence in support of the role of MicroRNA in adipogenesis. In general,the important roles of microRNA in the regulation of physiological processes such as energy balance are just beginning to be appreciated. In this regard, the findings of Micro-RNA-mediated regulation of adipogenesis are novel. Because the manuscript addresses the molecular mechanisms underlying the regulation of energy homeostasis, the findings are directly implicated in the control of body weight and thus prevention of overweight and obesity. This is of high medical impact particularly in developed Western countries. The technical strength is high, and the data are clear. The 3T3 cell system is a well-established model for studying white adipocyte differentiation. ob/ob, db/db and DIO are also adequate models in addressing in vivo roles.

Referee #3 (Remarks):

(1) Fig. 2C: As compared to the MOCK, the protein level of SIRT1 was apparently reduced by the treatment of the micro-RNA activator control (miR Ac CTL). Densitometry should be performed to quantify the results. From the gel shown, the control per se has strong effect on the SIRT1 protein level, which weakens the validity of using the activator in the study.

(2) Fig.2E: Similarly, the control of the micro-RNA inhibitor (miR In CTL) apparently upregulated the protein level of SIRT1. Densitometry of the protein should be performed to reflect the overall effects. Again, from the gel shown, the control itself seems to have a potent effect on SIRT1 protein level, which significantly weakens the validity of using this control in the studies.

(3) In Fig. 2, the numbers of treatments (i.e., the values of "n") are missing. This information must be provided in the legend. The statistical difference between the treated group, either activator-treated or inhibitor-treated, and the control group (CTL) should be presented.

(4)In Fig. 3B, why did the knockdown of SIRT1 (in MOCK group) not affect differentiation of the cells? This contradicts the observation on the effects exerted by the microRNA activator that downregulates SIRT1 levels and induces differentiation.

(5) In Fig. 3, the numbers of treatments (i.e., the values of "n") are missing. This information must be provided in the legend.

(6) Fig 4: The message level of FOXO1 does not alter in concert with that of the total protein level. The activator or the inhibitor appears to affect the message level without altering the total protein level of FOXO1. What does this tell? More mechanistic interpretation is needed.

(7) In the animal study part (Fig.5), a major concern is tissue specificity. Following i.p. injection, the major organ affected is usually liver. If this were the case, the interpretation would be changed. The weight loss effect shown in Fig.5C may have a significant contribution from the altered lipogenesis in liver. The in vivo part may not add additional strength to the entire conclusion drawn from the studies.

1st Revision - authors' response 23 June 2013

Response to Reviewers' Comments

We thank the Reviewers for their thoughtful and constructive reviews of our manuscript. We have attempted to address each of comments in the text and with additional experiments. Their valuable suggestions significantly improved the quality and clarity of our manuscript. Detailed point-by-point responses are provided below, with the Reviewers' comments indicated in italics.

Referee #1 (Comments on Novelty/Model System):
This ms finds a role for increasing miRNA146b with adipose differentiation (increase miRNA146b, decrease SIRT1, decreased FOXO1 deacetylation). This is interesting and novel. A major gap in the paper is the lack of understanding the effect of miRNA146b knockdown on glucose homeostasis. I would require this before accepting the paper; it will entail a major amount of new experimental work to comply.

We would like to thank the Reviewer for the constructive comments that helped to improve our manuscript.
We have performed significant additional experiments that examined the effect of miR-146b knockdown on glucose metabolism. Glucose- and insulin-tolerance tests showed that miR-146b knockdown results in improved insulin resistance (new Supporting Fig. S8A and B). Additionally, we measured the levels of insulin, leptin, and adiponectin in the blood and found that miR-146b knockdown effectively ameliorates hyperinsulinemia, hyperleptinemia, and HOMA-IR (new Supporting Table S4).

Referee #1 (Remarks):
This ms finds that miRNA146b increases with and promotes adipose development, while inhibiting it prevents adipose development. The miRNA binds to the 3'UTR of SIRT1 and manipulating miR146b affects SIRT1 RNA and protein (inc miR will dec SIRT1); miR146b acts via SIRT1 to affect adipose differentiation and FOXO1 is downstream. miR146b is increased in mouse obesity models and reduction of miR146b by LNA reduced adiposity in mice.
The observations are novel and interesting.
1. A major omission in the paper is examination of the effect that the LNA/reduced adiposity has on the physiology of the mice. Are these lean, healthy insulin-sensitive mice? Or are they lipodystrophic, insulin resistant? There is a lot of physiology that could/should be done to address this point. The paper would be hugely improved by inclusion of such data (eg clamp, lipid levels, etc).

We appreciate the Reviewer's comment that requested clarification of the physiological relevance of our LNA-miR146b antagomir data.
We have investigated this question in detail. We analyzed blood lipid profiles and found that LNA-miR146b injection significantly improved dyslipidemia (new Supporting Table S3). To identify whether miR-146b knockdown contributes to the amelioration of insulin resistance, we performed glucose- and insulin-tolerance tests. We observed increased glucose tolerance and insulin sensitivity in LNA-146b injected mice (new Fig. S8A and B). Blood hormone measurements indicated that the LNA-146b antagomir effectively reduced circulating insulin and leptin levels. HOMA-IR was also significantly inhibited in LNA-146b injected mice (new Supporting Table S4).
Together, these results suggest that miR-146b knockdown significantly enhanced insulin sensitivity in obese mice that are fed high-fat diets.

Referee #2 (Comments on Novelty/Model System):
The novelty of this work is significantly dampened by previous report that SIRT1 regulates adipogenesis and Sirt1+/- mice have reduction in fat. Identifying SIRT1 as a target of miR-146b is indeed a novel finding but authors did not produce convincing data to support a miR-146b/SIRT1 pathway regulates adipogenesis. This is partly due to the fact that their transfection system significantly affects the expression levels of SIRT1, one of their major targets. In addition, their characterization of miR-146b knockdown mice is quite superficial making it difficult to conclude that 146b regulates adipogenesis in vivo.

We wish to thank the Reviewer for the insightful critique. As the Reviewer pointed out, previous reports suggested an anti-adipogenic role of SIRT1 in white adipocytes and mesenchymal stem cells (Backesjo et al, 2006; Picard et al, 2004). However, the current report is the first finding that the miR-146b/SIRT1 pathway regulates adipogenesis.
To address the concern regarding our transfection system, we repeated miRNA functional analysis using the second generation of mirVana™ miRNA Mimic and Inhibitors (Life Technologies), which more specifically and potently targets miRNAs. Figures 1 (except Fig. 1A), Fig. 2 (except Fig. 2A and F), Fig. 3A-B, and Fig. 4. The corresponding legends and the manuscript text, have been modified accordingly, to highlight these new data.
To characterize the miR-146b knockdown mice, we analyzed their metabolic characteristics in detail, including blood lipid profiles (new Supporting Table S3), hormones (new Supporting Table S4), glucose- and insulin-tolerance tests (new Fig. S8), and immunoblotting of adipogenic markers (new Fig. 5F).
Thus, we have conducted extensive new physiological characterization and analyses of miR-146b knockdown mice, and have validated our original transfection method by repeating these critical experiments with an alternative transfection system, with similar results.

Referee #2 (Remarks):

The manuscript by Ahn et al reports that miR-146b level increased during 3T3-L1 differentiation, and inhibition or activation of miR-146b decreased or increased adipogenesis respectively. The authors further identified that miR-146b targets SIRT1 to increase FOXO1 acetylation, which mediates its pre-adipogenic effect. They then went on to suppress miR-146b in mice and demonstrated reduced adiposity and hepatosteatosis. In general, this article addresses a very interesting subject in metabolic regulation but the approach is very limited and the mechanism

proposed is only partially supported by the authors' data. Key information is missing for several experiments. Therefore, a significant number of additional experiments and clarifications are necessary for the manuscript to be convincing.

Major issues
1. In Fig. 2C, Sirt1 levels transfected with miR-146b Ac or Ac CTL were both significantly lower than that in mock transfection yet only miR-146b Ac induced adipogenesis as shown in Figure 1C. The authors should test different doses of Ac and CTL to make sure that altered SIRT1 expression and adipogenesis were not caused by the transfection per se. The same problem exists in Figure 2E but it is even more troubling since both transfections here increased SIRT1 expression as compared to MOCK. Therefore it appears that the current experimental system is not suitable for studying the role of SIRT1 in adipogenesis. Instead, a lentivirus or retrovirus based system should be used.

We are grateful for the Reviewer's constructive comments. To address the concern regarding our transfection system, we repeated miRNA functional analysis using newly released miRNA Mimic and Inhibitors from Life Technologies, which more specifically and potently targets miRNAs versus earlier systems. The new data obtained in these additional experiments have been incorporated to new Figures 1 (except Fig. 1A), new Fig. 2 (except Fig. 2A and F), new Fig. 3A-B, and new Fig 4. The corresponding legends and manuscript text have been modified accordingly. (Please see our response to *Comments on Novelty/Model System*, above).

2. Again in Figure 2C, SIRT1 protein level in miR Ac CTL transfection is clearly lower than that in MOCK but their mRNA levels are almost the same as shown in Figure 2B. Is there any post-transcription regulation here?

To eliminate the unintentional effects of miR Ac CTL or In CTL transfection on SIRT1 expression, we performed miRNA functional studies using improved experiment tools that have increased transfection specificity and potency. Thus we have validated that the miRNA activator negative control does not target any miRNA and downstream genes (new Fig. 2C).

3. Fig. 3A was clearly generated from two blots based on the misalignment of actin bands and different background so they cannot be compared.

As suggested, we have performed additional western blotting experiments and provide these data in new Supporting Figure S2A. These new immunoblots clearly indicate effective SIRT1 downregulation.

4. Foxo1 protein levels in cell lysate need to be shown before the IP data in Figure 4 can be appropriately interpreted. And this applies to Figure 5 as well.

To rule out inadvertent effects of miR Ac CTL or miR In CTL on FOXO1 expression, we changed our miRNA functional study tools and performed a new set of experiments. We have now measured acetylated FOXO1 protein expression using an anti-Ac-FOXO1 antibody (sc-49437, Santa Cruz Biotechnology). Using a new miR-146b specific activator or inhibitor, we have examined the effect of miR-146b on FOXO1 deacetylation *in vitro* (new Fig. 4). We also measured the level of acetylated FOXO1 in adipose tissue from miR-146b knockdown mice (new Fig. 5B).

5. The authors interpret that the reduced adiposity in miR-146b knockdown mice was caused by decreased adipocyte differentiation yet did not show a single adipogenic markers such as PPARgamma or aP2. In fact, SIRT1 has been showed to be able to regulate fatty acid oxidation in the liver. All physiological changes observed in miR-146b knockdown mice can be explained if reduced miR-146b in the liver increases SIRT1 level. All these possibilities have to be addressed before the authors can conclude that miR-146b regulates adipogenesis in vivo.

We have performed western blotting that demonstrates altered expression of adipogenesis-related factors such as C/EBPα, PPARγ, fatty acid synthase, and AP2 in white adipose tissue from miR-146b knockdown mice (new Fig. 5F).
The Reviewer has pointed out an important physiological role of hepatic SIRT1. Administration of LNA-miR-146b significantly increased SIRT1 levels and decreased fat accumulation in liver tissues

(new Fig. S7C and D). Our results are in agreement with a previous study (Li et al, 2011), in which systemic SIRT1 overexpression protected insulin resistance and improved metabolic parameters.
In contrast, hepatic-specific SIRT1 deletion showed greater lipid accumulation in the liver as a result of reduced fatty acid oxidation; however SIRT1 deletion does not alter serum triglyceride and cholesterol levels (Purushotham et al, 2009). Moreover, hepatic knockout of SIRT1 fails to alter serum insulin and leptin levels and shows normal insulin sensitivity and fuel metabolism in white adipose tissue and muscle. Thus, hepatic-specific SIRT1 knockout does not cause systemic glucose intolerance. These observations suggest that upregulation of hepatic SIRT1 alone is not sufficient to improve systemic insulin resistance, dyslipidemia, and hyperinsulinemia.
Instead, adipose tissue is important in controlling whole-body metabolism by releasing adipokines and storing free fatty acids. Thus, increased adipose mass via hyperplasia and hypertrophy results in insulin resistance and adipocyte dysfunction (Roberts et al, 2009). Therefore, reduced adiposity through downregulated miR-146b plays a crucial role in attenuating metabolic disorders including insulin resistance and dyslipidemia.
We have incorporated a detailed explanation into the Discussion section of our manuscript.

Minor issues
1. Critical physiological parameters such as the levels of serum lipids, glucose and key metabolic hormones in control and miR-146b knockdown mice need to be provided to establish a clear picture on the physiological changes in these mice.

We measured serum lipid profiles and metabolic parameters such as insulin, leptin, adiponectin, and fasting glucose levels (new Supporting Tables S3 and S4). We also analyzed systemic insulin resistance via glucose- and insulin-tolerance tests (new Fig. S8A and B).

Referee #3 (Comments on Novelty/Model System):

The authors provided evidence in support of the role of MicroRNA in adipogenesis. In general,the important roles of microRNA in the regulation of physiological processes such as energy balance are just beginning to be appreciated. In this regard, the findings of Micro-RNA-mediated regulation of adipogenesis are novel. Because the manuscript addresses the molecular mechanisms underlying the regulation of energy homeostasis, the findings are directly implicated in the control of body weight and thus prevention of overweight and obesity. This is of high medical impact particularly in developed Western countries. The technical strength is high, and the data are clear. The 3T3 cell system is a well-established model for studying white adipocyte differentiation. ob/ob, db/db and DIO are also adequate models in addressing in vivo roles.

We appreciate very much that the Reviewer found our work to be very interesting and of import. We thank the Reviewer for the helpful suggestions, for which we provide point-by-point responses.

Referee #3 (Remarks):

(1) Fig. 2C: As compared to the MOCK, the protein level of SIRT1 was apparently reduced by the treatment of the micro-RNA activator control (miR Ac CTL). Densitometry should be performed to quantify the results. From the gel shown, the control per se has strong effect on the SIRT1 protein level, which weakens the validity of using the activator in the study. (2) Fig.2E: Similarly, the control of the micro-RNA inhibitor (miR In CTL) apparently upregulated the protein level of SIRT1. Densitometry of the protein should be performed to reflect the overall effects. Again, from the gel shown, the control itself seems to have a potent effect on SIRT1 protein level, which significantly weakens the validity of using this control in the studies.

(1) and (2): To eliminate unintentional effects of miR Ac CTL or In CTL on SIRT1 expression, we performed new miRNA functional studies using the newly released miRNA Mimic and Inhibitors from Life Technologies, which are more specific and potent than previous products. Using this system, we have eliminated the inadvertent effects of miRNA negative controls on SIRT1 expression (new Fig. 2B - E).

(3) In Fig. 2, the numbers of treatments (i.e., the values of "n") are missing. This information must be provided in the legend. The statistical difference between the treated group, either activator-treated or inhibitor-treated, and the control group (CTL) should be presented.
We now show the numbers of treatments in all figure legends including Figure 2 legend. The statistical differences between the treated groups and control group are also indicated.

(4) In Fig. 3B, why did the knockdown of SIRT1 (in MOCK group) not affect differentiation of the cells? This contradicts the observation on the effects exerted by the microRNA activator that downregulates SIRT1 levels and induces differentiation.

The Reviewer is correct that MOCK and SIRT1 knockdown groups appear to be similar in the Oil Red O staining picture. We apologize for causing misunderstanding, which was due to the mistaken selection of representative photographs. We have added quantitative measurements of lipid accumulation in new Fig. 3B and substituted the photographs in Fig. 3A with new photos that better reflect the true effects of treatment.
Additionally, we have confirmed that SIRT1 knockdown resulted in significantly increased fat accumulation, as determined by Oil Red O staining after 8 days of differentiation (new Supporting Fig. S2A -C).

(5) In Fig. 3, the numbers of treatments (i.e., the values of "n") are missing. This information must be provided in the legend.

Now we show the numbers of treatments in all figure legends including Fig. 3 legend.

(6) Fig 4: The message level of FOXO1 does not alter in concert with that of the total protein level. The activator or the inhibitor appears to affect the message level without altering the total protein level of FOXO1. What does this tell? More mechanistic interpretation is needed.

To rule out inadvertent effects on FOXO1 by miR Ac CTL or miR In CTL, we changed our miRNA functional study tools and performed a new set of experiments.
In adipose tissue, FOXO1 interacts with PPARγ and negatively regulates its transcriptional activity (Dowell et al, 2003). Acetylation of FOXO1 attenuates DNA-binding and represses its transcriptional activity (Matsuzaki et al, 2005). SIRT1 regulates FOXO1 transactivation activity by deacetylating three lysine residues within the forkhead DNA binding domain (van der Heide & Smidt, 2005). Similarly, SIRT2 regulates 3T3-L differentiation by regulating FOXO1 acetylation/deacetylation. Therefore, measuring Ac-FOXO1 could provide more relevant information about the role of FOXO1, a downstream candidate of the miR-146b/SIRT1 pathway. Thus, we have performed new set of western blotting experiments with anti-Ac-FOXO1 antibody (sc-49437, Santa Cruz Biotechnology). New data are shown in Fig. 4A-D. The results suggest that FOXO1 is modulated downstream of the miR-146b/SIRT1 pathway.

(7) In the animal study part (Fig.5), a major concern is tissue specificity. Following i.p. injection, the major organ affected is usually liver. If this were the case, the interpretation would be changed. The weight loss effect shown in Fig.5C may have a significant contribution from the altered lipogenesis in liver. The in vivo part may not add additional strength to the entire conclusion drawn from the studies.

We understand your concern regarding tissue specificity. The administration of LNA-miR-146b significantly increased SIRT1 levels and decreased fat accumulation in liver tissues (Fig. S7). We characterized the metabolic type and found that the LNA-146b antagomir effectively improved metabolic disorders such as insulin resistance and dyslipidemia in high fat-fed mice similar to SIRT1 overexpression (new Supporting Tables S3 and S4, and new Supporting Fig. S8). Our results agree with previous findings (Li et al, 2011), in which systemic SIRT1 overexpression protected against insulin resistance and improved metabolic parameters.
Interestingly, hepatic-specific deletion of SIRT1 shows greater lipid accumulation in the liver as a result of reduced fatty acid oxidation; However, this does not alter serum triglyceride and cholesterol levels (Purushotham et al, 2009). Moreover, hepatic knockout of SIRT1 fails to alter serum insulin and leptin levels and shows normal insulin sensitivity and fuel metabolism in white adipose tissue and muscle; thus these animals do not develop systemic glucose intolerance. These

results indicate that upregulation of hepatic SIRT1 is not sufficient to improve systemic insulin resistance, dyslipidemia, and hyperinsulinemia.
On the other hand, adipose tissue is important in controlling whole body metabolism by releasing adipokines and storing free fatty acids. Thus, increased adipose mass via hyperplasia and hypertrophy results in insulin resistance and adipocyte dysfunction (Roberts et al, 2009). Collectively, reduced adiposity through downregulated miR-146b plays a crucial role in attenuating metabolic disorders including insulin resistance and dyslipidemia. We have incorporated additional detailed explanation into the Discussion section.
The authors are extremely appreciative of the time and effort that the *EMBO Molecular Medicine* reviewers and editorial staff have provided in thoroughly assessing our report. We believe that we have satisfactorily addressed all critiques and queries, and have performed a significant number of new experiments that confirm our initial conclusions. We are confident that our current manuscript is now of the quality, clarity, and depth required for publication in *EMBO Molecular Medicine*, and will await your editorial decision regarding publication of our revised manuscript.

References
Backesjo CM, Li Y, Lindgren U, Haldosen LA (2006) Activation of Sirt1 decreases adipocyte formation during osteoblast differentiation of mesenchymal stem cells. Journal of bone and mineral research : the official journal of the American Society for Bone and Mineral Research 21: 993-1002

Dowell P, Otto TC, Adi S, Lane MD (2003) Convergence of peroxisome proliferator-activated receptor gamma and Foxo1 signaling pathways. The Journal of biological chemistry 278: 45485-45491

Li Y, Xu S, Giles A, Nakamura K, Lee JW, Hou X, Donmez G, Li J, Luo Z, Walsh K et al (2011) Hepatic overexpression of SIRT1 in mice attenuates endoplasmic reticulum stress and insulin resistance in the liver. FASEB journal : official publication of the Federation of American Societies for Experimental Biology 25: 1664-1679

Matsuzaki H, Daitoku H, Hatta M, Aoyama H, Yoshimochi K, Fukamizu A (2005) Acetylation of Foxo1 alters its DNA-binding ability and sensitivity to phosphorylation. Proceedings of the National Academy of Sciences of the United States of America 102: 11278-11283

Picard F, Kurtev M, Chung N, Topark-Ngarm A, Senawong T, Machado De Oliveira R, Leid M, McBurney MW, Guarente L (2004) Sirt1 promotes fat mobilization in white adipocytes by repressing PPAR-gamma. Nature 429: 771-776

Purushotham A, Schug TT, Xu Q, Surapureddi S, Guo X, Li X (2009) Hepatocyte-specific deletion of SIRT1 alters fatty acid metabolism and results in hepatic steatosis and inflammation. Cell metabolism 9: 327-338

Roberts R, Hodson L, Dennis AL, Neville MJ, Humphreys SM, Harnden KE, Micklem KJ, Frayn KN (2009) Markers of de novo lipogenesis in adipose tissue: associations with small adipocytes and insulin sensitivity in humans. Diabetologia 52: 882-890

van der Heide LP, Smidt MP (2005) Regulation of FoxO activity by CBP/p300-mediated acetylation. Trends in biochemical sciences 30: 81-86

2nd Editorial Decision 11 July 2013

Thank you for the submission of your revised manuscript to EMBO Molecular Medicine. We have now received the enclosed reports from the referees that were asked to re-assess it. As you will see the reviewers are now globally supportive and I am pleased to inform you that we will be able to accept your manuscript pending the following final amendments:

As you can see, referee 2 agrees that the data shows in vitro that he miR146b-Sirt1-Foxo1 pathway regulates adipogenesis, and in vivo that systemic inhibition of miR146b improves glucose and lipid metabolism. However, this referee is still not convinced that the axis is shown to control adipogenesis in mice. As such, while at this point I will not ask you to perform any further experiments, unless you already have data at hand I would greatly appreciate if you could address the issue raised in writing and reflect the referee's concern in the results and discussion section of the manuscript.

I have noticed that the manuscript does not contain a microarray section in the materials and methods. It is essential to the global understanding that all experiments are described in the main body of the manuscript including microarray accession numbers. Please make sure that all experiments are described in the material and methods (including the microarrays source, analysis, data mining and the bioinformatics search).

Data of gene expression experiments described in submitted manuscripts should be deposited in a MIAME-compliant format with one of the public databases. We would therefore ask you to submit your microarray data to the ArrayExpress database maintained by the European Bioinformatics Institute for example. ArrayExpress allows authors to submit their data to a confidential section of the database, where they can be put on hold until the time of publication of the corresponding manuscript. Please see http:www.ebi.ac.uk/arrayexpress/Submissions/ or contact the support team at arrayexpress@ebi.ac.uk for further information.

Please submit your revised manuscript within two weeks.

I look forward to reading a new revised version of your manuscript as soon as possible.

***** Reviewer's comments *****

Referee #1 (Remarks):

As noted previously, this ms finds that miRNA146b increases with and promotes adipose development, while inhibiting it prevents adipose development. The miRNA binds to the 3'UTR of SIRT1 and manipulating miR146b affects SIRT1 RNA and protein (inc miR will dec SIRT1); miR146b acts via SIRT1 to affect adipose differentiation and FOXO1 is downstream. miR146b is increased in mouse obesity models and reduction of miR146b by LNA reduced adiposity in mice. The revised ms now also shows that the decreased adiposity is accompanied by improved glucose/lipid homeostasis. Additionally the revised ms presents much clearer, more convincing data that the various interventions really achieved the desired effects on miRNA146b and Sirt1 levels.

The authors have responded nicely to the other reviewers and my comments.

Referee #2 (Comments on Novelty/Model System):

Systemic inhibition of 146b in mice by LNA-miR-146b cannot convincingly establish that the proposed miR146b-SIRT1-FOXO1 pathway regulates adipogenesis in vivo.

Referee #2 (Remarks):

The authors have addressed majority of my technical questions and the quality of the work has been significantly improved. However the authors failed to address my concern that "All physiological changes observed in miR-146b knockdown mice can be explained if reduced miR-146b in the liver increases SIRT1 level" and did not provide sufficient data to support that their working model of regulation of adipogenesis by miR146b-SIRT1-FOXO1 pathway is physiologically and medically relevant.

In the revised manuscript, the authors reported that administration of LNA-miR-146b indeed increase SIRT1 in the liver of mice but argued that "upregulation of hepatic SIRT1 alone is not sufficient to improve systemic insulin resistance, dyslipidemia, and hyperinsulinemia". Their assumption was largely based on information inferred from one particular report (Cell Metab. 2009 Apr;9(4):327-38.). But they failed to cite several publications (PNAS, 2007 Jul 31;104 (31):12861-6 and J Clin Invest. 2011 Nov; 121(11):4477-90.) showing that hepatic Sirt1 depletion alters systemic glucose and lipid metabolism.

In addition, the authors' approach systemically reduced 146b in most tissues in mice (Fig. S5A). If the miR146b-SIRT1 pathway acts in all tissues, this will lead to systemic activation of SIRT1, which has been reported to increase fatty acid oxidation and improved metabolic profile (Cell Metab. 2008 Nov;8(5): 347-58 and PNAS 2008 Jul 15;105(28):9793-8). An overall increase in fatty acid oxidation could deplete lipids in adipocytes and reduce expression levels of adipocyte-specific proteins. Therefore I think that the authors' data support that miR146b-SIRT1-FOXO1 pathway regulates adipogenesis in vitro, and their systemic inhibition of miR146b improved glucose and lipid metabolism in mice. But it is unclear how much the altered adiposity in vivo can be attributed to the specific pathway the authors have proposed. In my opinion this can only be resolved by performing experiments in mice with adipose-specific knockout or knockdown of miR146b.

Referee #3 (Comments on Novelty/Model System):

My concerns have all been well addressed. I am impressed by the authors' effort in performing extra experiments to improve the quality of their manuscript. The novelty remains high, and the medical impact remains high as the situations of obesity and the related insulin resistance are not improving.

Referee #3 (Remarks):

This is a piece of nicely revised work. It would bring up the attention of both molecular biologists and clinical scientists.

2nd Revision - authors' response 31 July 2013

Response to Editorial Comments

Please find enclosed revised manuscript “MicroRNA-146b promotes adipogenesis by suppressing the SIRT1-FOXO1 cascade”. We thank the Reviewers for their helpful comments. Their valuable suggestions significantly improved the quality and clarity of our manuscript. We also profoundly appreciate you for the help and understanding with our work.
We have incorporated the requested changes and provided our response to the Editorial comments, with the comments indicated in italics.

1) As you can see, referee 2 agrees that the data shows in vitro that the miR146b-Sirt1-Foxo1 pathway regulates adipogenesis, and in vivo that systemic inhibition of miR146b improves glucose and lipid metabolism. However, this referee is still not convinced that the axis is shown to control adipogenesis in mice. As such, while at this point I will not ask you to perform any further experiments, unless you already have data at hand I would greatly appreciate if you could address the issue raised in writing and reflect the referee's concern in the results and discussion section of the manuscript.

We understand the Reviewer #2’s concern and suggest the needs for further study in *Discussion* as below.
Consistently, the study by Erion et al. (PNAS, 2009, 106:11288-11293) reported that decrease of SIRT1 in liver and adipocyte effectively reduced white adipose tissue mass and improved glucose tolerance. Although we found systemic knockdown of miR-146b effectively reduced body weight and adiposity, further study needs to confirm the direct role of miR-146b using by adipose tissue-specific knockout of miR-146b

2) I have noticed that the manuscript does not contain a microarray section in the materials and methods. It is essential to the global understanding that all experiments are described in the main body of the manuscript including microarray accession numbers. Please make sure that all experiments are described in the material and methods (including the microarrays source, analysis, data mining and the bioinformatics search).

We performed miRNA microarray analysis to search interesting miRNAs which were significantly changed by differentiation stimuli. Though the data was now shown, we explained how to perform miRNA microarray anlaysis in the *'Supplementary Materials and Method'* section. We submitted our array raw data file to *ArrayExpress* website according to your suggestion. The accession number is E-MTAB-1783.

3) *Data of gene expression experiments described in submitted manuscripts should be deposited in a MIAME-compliant format with one of the public databases. We would therefore ask you to submit your microarray data to the ArrayExpress database maintained by the European Bioinformatics Institute for example. ArrayExpress allows authors to submit their data to a confidential section of the database, where they can be put on hold until the time of publication of the corresponding manuscript. Please see http:www.ebi.ac.uk/arrayexpress/Submissions/ or contact the support team at arrayexpress@ebi.ac.uk for further information.*

We submitted our microRNA microarray data to ArrayExpress DB according to your suggestion. The accession number is E-MTAB-1783. We ask the delay of the public release date of the experiment until the publication of our manuscript.

© EMBO
