# Supplementary material for: MicroRNA-146b promotes adipogenesis by suppressing the SIRT1-FOXO1 cascade
Source: EMBO Mol Med. 2013 Sep 6;5(10):1602–12. doi: 10.1002/emmm.201302647 (PMC3799582; doi:10.1002/emmm.201302647)
Supplement: Supplementary file 2 [file emmm0005-1602-SD2.pdf]

Supporting Information

MicroRNA-146b promotes adipogenesis by suppressing the SIRT1-FOXO1 cascade

Authors: Jiyun Ahn, Hyunjung Lee, Chang Hwa Jung, Tae Il Jeon, and Taeyoul Ha

Table of Contents

Supplementary Materials and Methods 2

Supporting Table S1 4

Supporting Fig. S1 5

Supporting Fig. S2 6

Supporting Fig. S3 7

Supporting Table S2 8

Supporting Fig. S4 9

Supporting Fig. S5 10

Supporting Fig. S6 11

Supporting Table S3 12

Supporting Fig. S7 13

Supporting Fig. S8 14

Supporting Table S4 15

Supplementary Materials and Methods

MiRNA microarray analysis

MiRNA microarray analysis was used to obtain miRNA expression profiles of undifferentiated- and differentiated 3T3-L1 cells. Total RNA was extracted using RNeasy kit according to the protocol of the manufacturer (Qiagen, Valencia, CA). The RNA quantity was determined using Nanodrop1000 (Thermo Fisher Scientific, Waltham, MA) and the RNA quality was assessed using the Agilent 2100 Bioanalyzer (Agilent, Santa Clara, CA). RNA (100 ng) was amplified and labeled using the miRNA Complete Labeling and Hyb Kit (Agilent) according to the manufacturer's instructions. Cy3-labelled RNA was hybridized to Agilent Mouse miRNA Microarray 8x60K v17.0. Image scanning was performed using an Agilent C Scanner (Agilent G2505C) and microarray data were extracted using Agilent Feature Extraction v10.7.3.1 software. Data was analyzed by GeneSpring GX 11.5.1 (Agilent). The microarray data cited in this article were deposited in the ArrayExpress database (http://www.ebi.ac.uk/arrayexpress/) under accession number E-MTAB-1783.

Blood biomarker assay

Mice injected with LNA-miR-146b or LNA-scrambled control were sacrificed after a 12-h fast. Serum alanine aminotransferase (ALT) and aspartate aminotransferase (AST) levels were measured on a spectrophotometer with a kit purchased from Shin Yang Diagnostics (Seoul, Korea). Serum triglyceride, cholesterol, HDLC-C, NEFA, and glucose were measured using commercially available kits (Shin Yang Diagnostics). Insulin, leptin, and adiponectin were measured by ELISA (ALPCO Diagnostics, Salem, NH).

Glucose and insulin tolerance test

Intraperitoneal glucose tolerance tests (IPGTT) was determined in response to intraperitoneal administration of 2 g D-glucose/kg body weight following a 4 h fast. Blood glucose was measured from the tail vein 0, 15, 30, 60, 90, and 120 minutes after glucose administration. Intraperitoneal insulin tolerance tests (IPITT) was determined in response to intraperitoneal administration of 1.2 IU human insulin/kg body weight following a 4 h fast. Blood glucose was measured 0, 15, 30, 60, 90, and 120 minutes after insulin administration.

Histological examination of livers

Liver tissues were fixed in 10% buffered formalin, embedded in paraffin, sectioned, and stained with

hematoxylin and eosin for histological studies. Stained areas were observed with a light microscope (Olympus, Tokyo, Japan) at 200× magnification.

Supporting Table S1. Changes in the expression levels of various miRNAs after adipogenesis in 3T3-L1.

	MicroRNA	Mean	SD
Downregulation	miR 139-3p	0.03*	0.01
	miR 221	0.35*	0.02
	miR 297b-5p	0.58*	0.01
	miR 302a	0.56*	0.03
	miR 324-5p	0.32*	0.01
	miR 466F-5p	0.57*	0.01
	miR 468	0.46*	0.26
	miR 720	0.12*	0.01
Upregulation	miR 143	1.27*	0.02
	miR 146b	9.01*	0.04

*P < 0.05 versus undifferentiated 3T3-L1 cells

Fig. S1

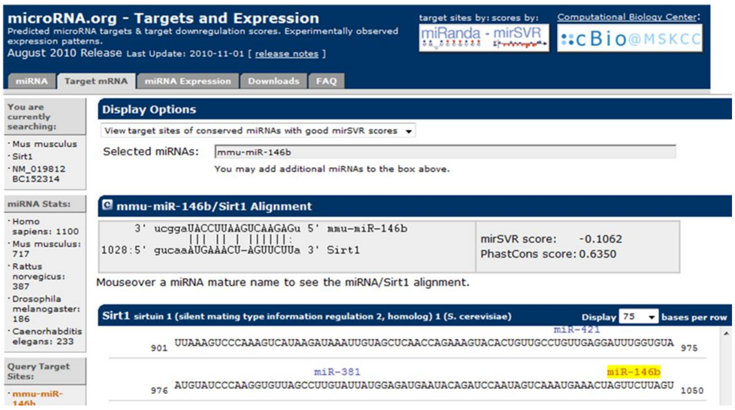

Fig. S1. Target analysis predicts that SIRT1 is a potential target of miR-146b.

MicroRNA target prediction databases, such as microRNA.org, showed alignment between miR-146b and SIRT1.

Fig. S2

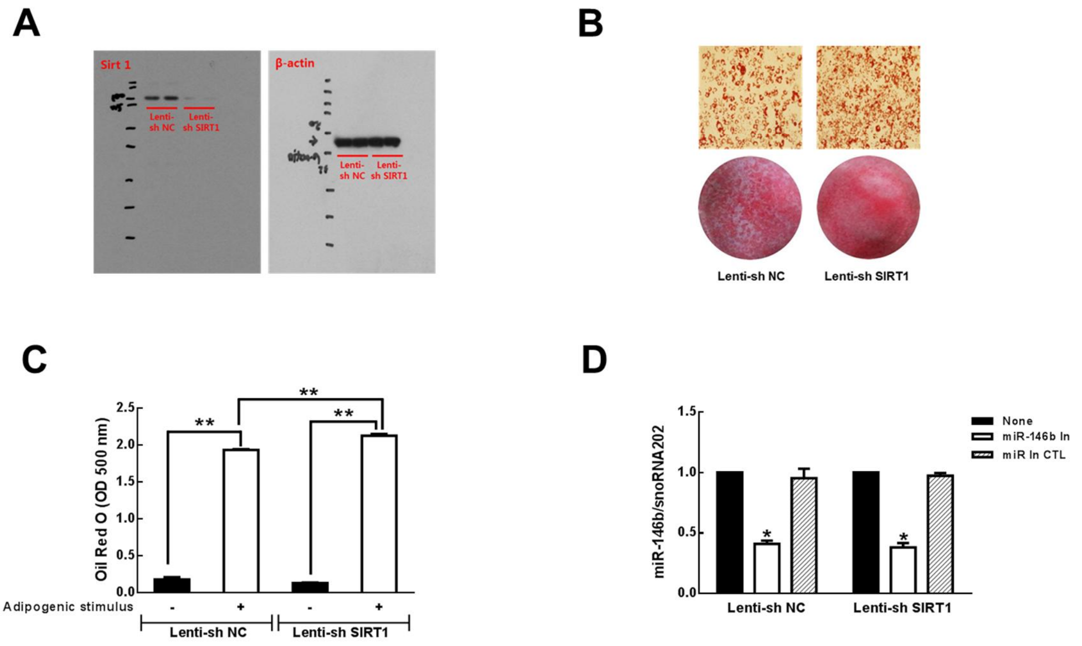

Fig. S2. Knockdown of SIRT1 increased lipid accumulation in differentiated 3T3-L1 cells.

A. SIRT1 was knocked down by lentiviral shRNA-SIRT1 (Lenti-sh SIRT1) and confirmed by western blot analysis. Lenti-sh NC, lentiviral shRNA negative control.

B. Preadipocytes transduced with Lenti-sh NC- or Lenti-sh were stimulated to differentiation. At day 8, differentiated cells were stained with Oil red O.

C. Intracellular lipid accumulation was quantified by measuring optical absorbance at 500 nm (n=3). ***P* < 0.01. Values are means ± SD.

D. Preadipocytes that were transduced with Lenti-sh NC or Lenti-sh SIRT1 were transfected with miR-146b In or miR In CTL and stimulated to differentiate. Expression levels of miR-146b and SIRT1 were quantified by qRT-PCR on day 8 of differentiation (n=3). Values are means ± SD. **P* < 0.05 versus miR In CTL.

Fig. S3

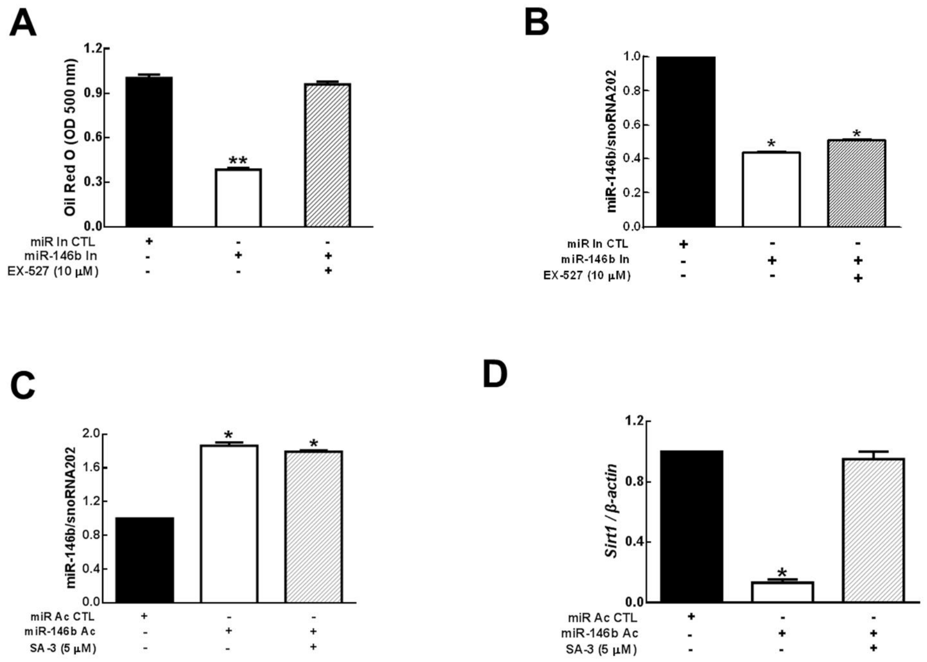

Fig. S3. SIRT1 mediates the adipogenic effect of miR-146b in 3T3-L1 cells.

A. The effect of EX-527 on the anti-adipogenic activity of miR-146b In. Cells were pretreated with 10 µM EX-527 for 6 hours. Media were then replaced with differentiation media containing 10 µM EX-527. Cells were stained with Oil red O on day 8 of differentiation, and lipid droplets were quantified (n=3). Values are means ± SD. *$P < 0.05$ versus miR In CTL.

B. The effect of EX-527 on miR-146b expression in the experiment described in (A). Expression of miR-146b was measured by qRT-PCR (n=3). Values are means ± SD. *$P < 0.05$ versus miR In CTL.

C. The effect of SA-3 on miR-146b expression. 3T3-L1 cells were transfected with miR-146b Ac or miR-Ac CTL. After 2 days, cells were maintained in growth media containing 5 µM SA-3 for 8 days. Expression levels of miR-146b were measured by qRT-PCR (n=3). Values are means ± SD. *$P < 0.05$ versus miR Ac CTL.

D. The effect of SA-3 on SIRT1 mRNA expression in the experiment described in (C). Expression levels of SIRT1 mRNA were measured by qRT-PCR (n=3). Values are means ± SD. *$P < 0.05$ versus miR Ac CTL.

Supporting Table S2. Measurement of body weight and white adipose tissue (WAT) weight

(g)	1		2		3	
	WT	ob/ob	Lean	db/db	Chow	DIO
Body weight	28.5 ± 1.5	38.39 ± 0.9*	33.39 ± 1.89	53.94 ± 1.63*	26.48 ± 0.85	34.33 ± 1.75*
WAT weight	0.74 ± 0.23	2.24 ± 0.15*	0.87 ± 0.28	2.87 ± 0.39*	0.72 ± 0.19	1.51 ± 0.29*

Body weight and epididymal fat weight were measured. Data shows the mean ± SEM. n=8

* $P < 0.05$ versus matching control

WT, wild-type; Lean, lean control; Chow, chow-fed mice; DIO, diet-induced obese mice

Fig. S4

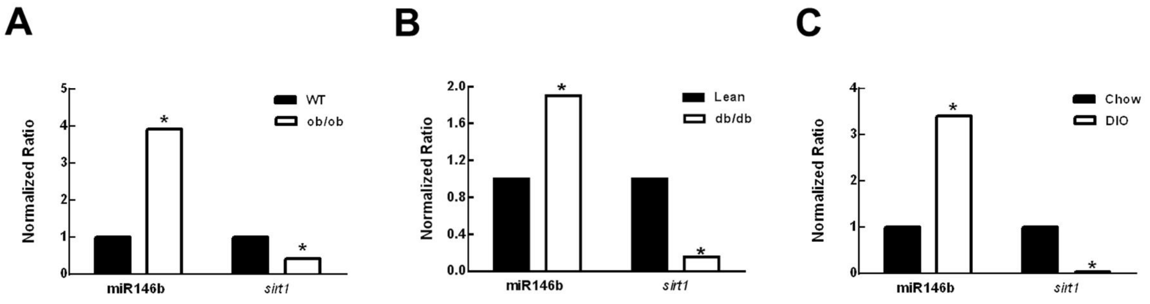

Fig. S4. Expression of miR-146b and its direct target, SIRT1 in WAT from various obese mice.

A-C. The increase in miR-146b and the resulting decrease in SIRT1 are correlated with hypertrophy of adipose tissue from obese mice, such as ob/ob (A), db/db (B), and diet-induced obese mice (C). qRT-PCR analysis was performed to measure miR-146b and SIRT1 mRNA expression levels in epididymal adipose tissue (n=5). Values are means ± SEM. *$P < 0.05$ versus corresponding control mice.

Fig. S5

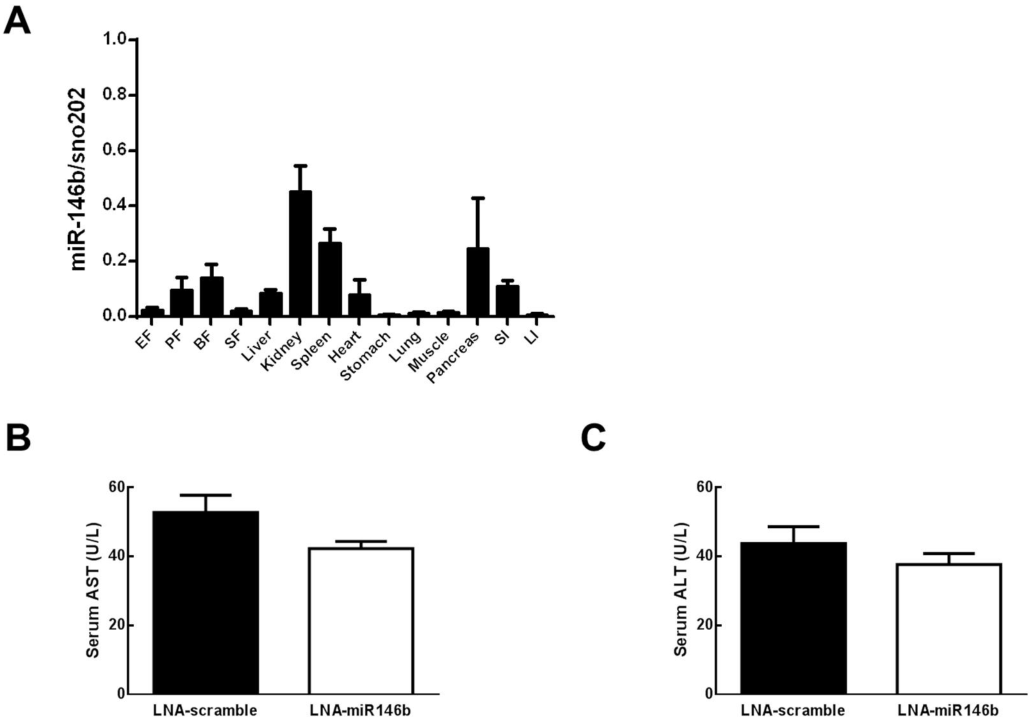

Fig. S5. Efficacy and toxicity of LNA-miR-146b antagomir.

A. Expression levels of miR-146b were measured by qRT-PCR in various tissues to test the efficacy of LNA-miR-146b antagomir in mice. Expression of miR-146b was compared between mice treated with LNA-miR-146b and those treated with LNA-scrambled negative control (n=5). Values are means ± SEM. EF, epididymal fat pad; PF, perirenal fat; BF, brown fat; SF, subcutaneous fat; SI, small intestine; LI, large intestine.

B-C. Effect of LNA-miR146b injection on AST and ALT, which are biomarkers of liver injury (n=5). Values are means ± SEM.

Fig. S6

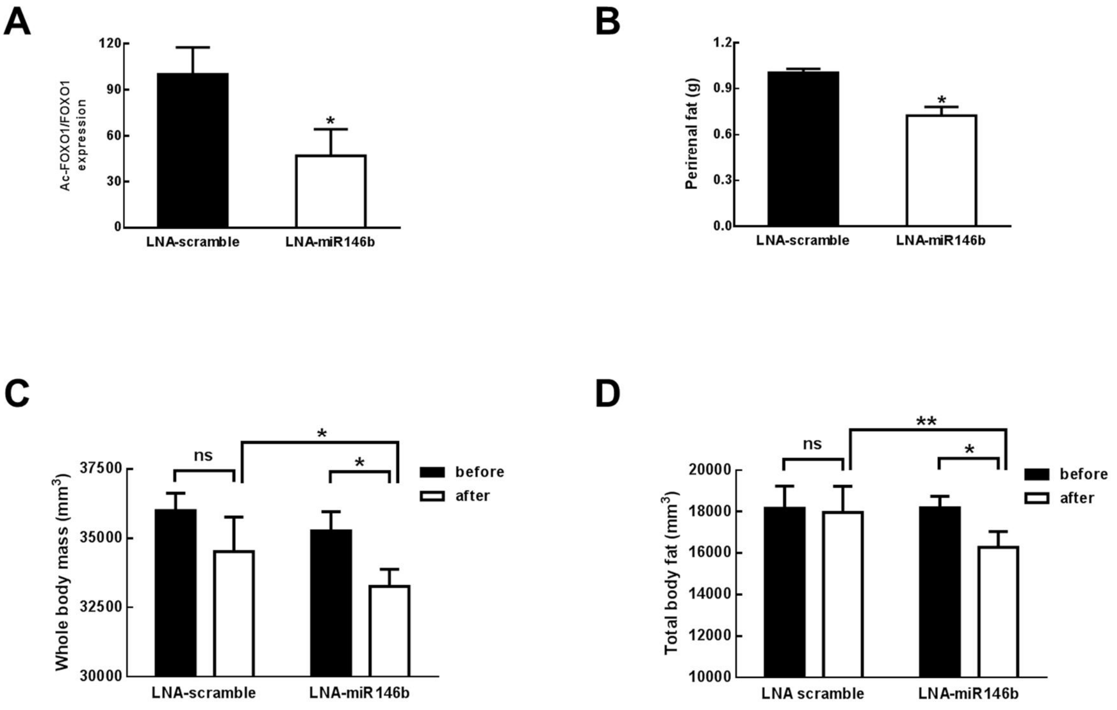

Fig. S6. Knockdown of miR-146b reduced adiposity and whole body mass in high fat-fed obese mice.

A. Knockdown of miR-146b by LNA-miR-146b significantly decreased the acetylated FOXO1(Ac-FOXO1)/total FOXO1 ratio of white adipose tissue. Ac-FOXO1/ FOXO1 ratio was based on densitometric quantifications of Ac-FOXO1 and total FOXO1 levels on Western blots (n=5). Values are means ± SEM. *P < 0.05 versus LNA-scramble.

B. Knockdown of miR-146b by LNA-miR-146b reduced white adipose fat mass. The weight of perirenal white adipose tissue was measured 72 h after the last injection (n=5). Values are means ± SEM. *P < 0.05 versus LNA-scramble.

C. Administration of LNA-miR-146b inhibitor (LNA-miR-146b) significantly decreased whole body mass (n=5). Values are means ± SEM. ns, not significant; *P < 0.05.

D. LNA-miR-146b significantly reduced total body fat mass compared to LNA-scramble (n=5). Values are means ± SEM. ns, not significant; *P < 0.05.

Supporting Table S3. Serum lipids profile in LNA-injected mice

	LNA-scramble (n=6)	LNA-miR-146b (n=6)
Triglyceride (mg/dL)	84.33 ± 8.59	43.19 ± 14.76 *
Total cholesterol (mg/dL)	187.4 ± 6.39	138.43 ± 9.35 *
HDL-C (mg/dL)	93.97 ± 8.99	55.57 ± 28.7 *
LDL-C (mg/dL)	83.74 ± 13.73	47.17 ± 20.87 *
NEFA (μEq/L)	1303.44 ± 65.38	835.45 ± 52.79 *

Mean±SEM, Significantly different with LNA-scramble. *$P < 0.05$
HDL-C, high density lipoprotein cholesterol; LDL-C, low density lipoprotein cholesterol, NEFA, non-esterfied fatty acids

Fig. S7

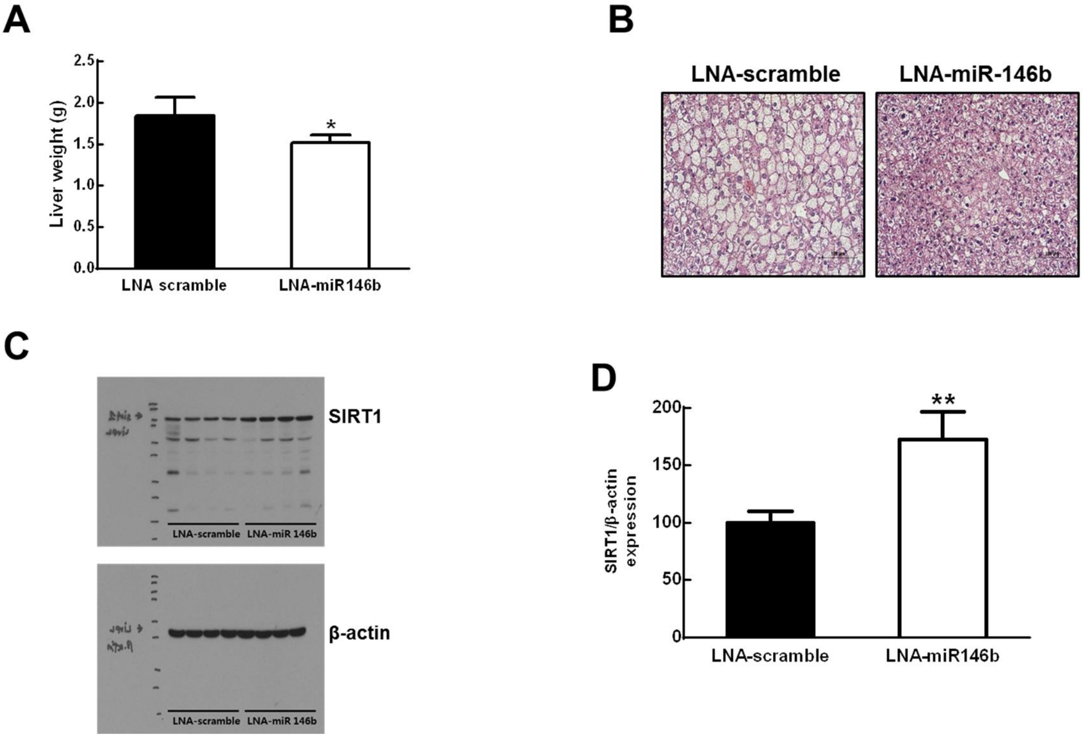

Fig. S7. Knockdown of miR-146b improved hepatic steatosis via upregulation of SIRT1 in high fat-fed obese mice.

A. Administration of LNA-miR-146b reduced hepatic hypertrophy (n=5). Values are means ± SEM. *$P < 0.05$ versus LNA-scramble.

B. Injection of LNA-miR-146b decreased intracellular lipid accumulation in hepatocytes. Hepatic tissues were stained with H&E for histological examination. Scale bar=100 μm.

C. Immunoblot showing SIRT1 and β-actin expressions.

D. Densitometric analyses for (C). Values are means ± SD. ** $P < 0.01$.

Fig. S8

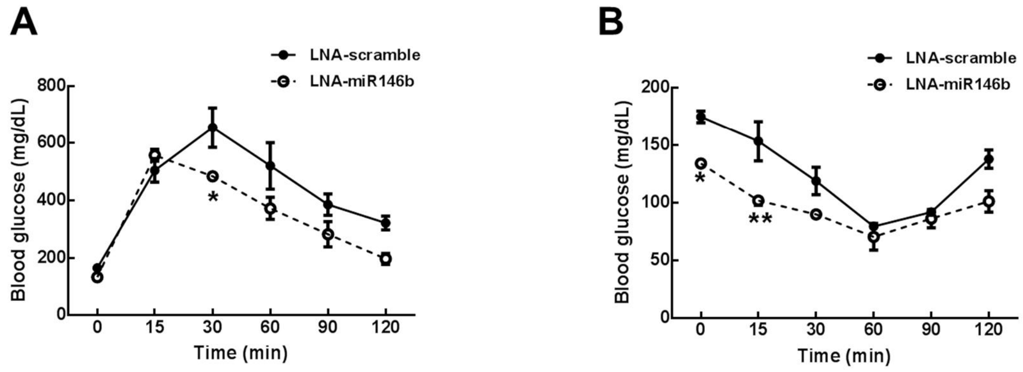

Fig. S8. Knockdown of miR-146b ameliorated insulin resistance in high fat-fed obese mice.

A. Glucose tolerance test in LNA-scramble and LNA-miR146b injected mice (n=6). Values are means ± SEM. *$P < 0.05$ versus LNA-scramble.

B. Insulin tolerance test in LNA-scramble and LNA-miR146b injected mice (n=6). Values are means ± SEM. *$P < 0.05$ versus LNA-scramble.

Supporting Table S4. Blood glucose and hormones levels in LNA-injected mice

	LNA-scramble (n=6)	LNA-miR-146b (n=6)
FBG (mg/dL)	169.7 ± 8.4	132.9 ± 5.9 *
Insulin (ng/mL)	13.87 ± 0.13	5.37 ± 0.50*
Leptin (ng/mL)	32.57 ± 2.53	20.41 ± 0.92*
Adiponectin (μg/mL)	29.50 ± 4.84	31.36 ± 2.79
HOMA-IR	110.6 ± 5.3	34.7 ± 1.5*

Mean±SEM, Significantly different with LNA-scramble $*P < 0.05$
